# Synaptic pruning mechanisms and application of emerging imaging techniques in neurological disorders

**DOI:** 10.4103/NRR.NRR-D-24-01127

**Published:** 2025-04-29

**Authors:** Yakang Xing, Yi Mo, Qihui Chen, Xiao Li

**Affiliations:** 1 Key Laboratory of Prevention and Treatment of Cardiovascular and Cerebrovascular Diseases of Ministry of Education, Gannan Medical University, Ganzhou, Jiangxi Province, China; 2 Institute for Department of Physiology, School of Basic Medical Sciences, Gannan Medical University, Ganzhou, Jiangxi Province, China; 3 Department of Pathology, Anyang Maternal and Child Health Hospital, Anyang, Henan Province, China; 4 Ganzhou Key Laboratory of Neuroinflammation Research, Gannan Medical University, Ganzhou, Jiangxi Province, China; 5 Jiangxi Province Key Laboratory of Pharmacology of Traditional Chinese Medicine, Gannan Medical University, Ganzhou, Jiangxi Province, China

**Keywords:** chemokine, complement, experience-dependent driven synaptic pruning, imaging techniques, neuroglia, signaling pathways, synapse elimination, synaptic pruning

## Abstract

Synaptic pruning is a crucial process in synaptic refinement, eliminating unstable synaptic connections in neural circuits. This process is triggered and regulated primarily by spontaneous neural activity and experience-dependent mechanisms. The pruning process involves multiple molecular signals and a series of regulatory activities governing the “eat me” and “don’t eat me” states. Under physiological conditions, the interaction between glial cells and neurons results in the clearance of unnecessary synapses, maintaining normal neural circuit functionality via synaptic pruning. Alterations in genetic and environmental factors can lead to imbalanced synaptic pruning, thus promoting the occurrence and development of autism spectrum disorder, schizophrenia, Alzheimer’s disease, and other neurological disorders. In this review, we investigated the molecular mechanisms responsible for synaptic pruning during neural development. We focus on how synaptic pruning can regulate neural circuits and its association with neurological disorders. Furthermore, we discuss the application of emerging optical and imaging technologies to observe synaptic structure and function, as well as their potential for clinical translation. Our aim was to enhance our understanding of synaptic pruning during neural development, including the molecular basis underlying the regulation of synaptic function and the dynamic changes in synaptic density, and to investigate the potential role of these mechanisms in the pathophysiology of neurological diseases, thus providing a theoretical foundation for the treatment of neurological disorders.

## Introduction

During brain development, neurons migrate to specific locations and establish precise connections, thus forming the complex functional circuits of the central nervous system. As the brain regulates mental and behavioral activities, neuronal axons and dendrites extend to surrounding neurons or neural cells, forming structures known as synapses. Thus, synapses are crucial structures for the transmission of information between neural cells. The functional homeostasis of synapses is influenced by various factors, including neurotransmitter release and uptake, the strength and range of the postsynaptic response, and neuronal excitability (Hauswirth et al., 2018). The interactions between synapses (tripartite, tetrapartite, or penta-partite), otherwise known as synaptic connections, not only affect the transmission of information but also regulate the formation and remodeling of neural circuits (De Luca et al., 2020). However, traditional synaptic connections cannot explain the specific mechanisms by which the function and plasticity of synapses are influenced when they come into contact with other cells or substances. Therefore, there is a clear need for the development of an integrated, dynamic systems biology synaptic model based on molecular networks and interactions between cellular and subcellular components (Chanaday and Kavalali, 2022; Rizo, 2022). Tripartite synapses, which are composed of glial cells, influence neuronal activity by regulating internal calcium signaling, which in turn triggers the release of neurotransmitters to modulate synaptic transmission (Araque et al., 1999). Based on tripartite synapses, a tetrapartite synapse model includes the non-cellular component of the extracellular matrix (ECM), which forms a structural scaffold around the soma and synapses, thus creating a physical barrier that can objectively influence the spatiotemporal characteristics of synaptic transmission. Furthermore, components of the ECM can alter synaptic excitability and transmission efficiency by influencing ion channels and neurotransmitter receptors, thereby affecting information processing by the entire neural network (Dityatev et al., 2006). A more systematic synaptic connection model, the penta-partite synapse, focuses on the ECM and the neurovascular unit. This model incorporates metabolism, immunity, the blood‒brain barrier and lymphatic circulation, covering almost all factors that can influence synaptic function based on other synaptic connection components (De Luca et al., 2020). As research on synaptic connection patterns has advanced, this holistic, multi-component concept provides a more comprehensive framework for investigating synaptic function and neural circuits, as well as providing perspectives for studying mechanisms under pathological physiological conditions in the central nervous system.

Abnormal synaptic connections can lead to weakened synaptic transmission efficiency and strength, thus resulting in the development of corresponding neurological disorders. Consequently, the elimination of abnormal synaptic connections is crucial for maintaining synaptic homeostasis during the refinement of the central nervous system. Synaptic pruning is a typical clearance method that enhances synaptic connections by selectively eliminating unnecessary synapses via neuroglial cells, thereby modifying neural network connection patterns (Purves and Lichtman, 1980). This is also a key link in the interaction between neuroglia and neurons (Wilton et al., 2019). However, questions remain with respect to how the brain initiates synaptic pruning and the specific mechanisms that can regulate this process. The level of neuronal activity may be one of the triggers for synaptic pruning, whereas synaptic scaling adjusts the synaptic strength to maintain overall neuronal excitability when neuronal activity continuously increases or decreases. A-amino-3-hydroxy-5-methyl-4-isox-azolepropionic acid receptor (AMPAR) is one of the key mechanisms triggering synaptic pruning and is also the main effector of synaptic scaling. Synaptic scaling is a process that can alter synaptic strength by regulating the number and subunits of AMPAR (Barnes et al., 2017). A previous meta-analysis (Moulin et al., 2022) reported that the induction of synaptic scaling reduced the number of dendritic spines by specific synaptic downscaling to protect the overall homeostasis of the neural network, such as reducing the number of spines in epilepsy to reduce neuronal hyperexcitability. Conversely, sensory deprivation leads to neuronal inactivation; under these conditions, synaptic scaling tends to increase the volume rather than the number of dendritic spines, whereas the reduction in the number of dendritic spines may be mediated by NMDAR-dependent pruning following inactivation, possibly to protect normal synapses from being excessively pruned. For example, following sensory deprivation, synaptic scaling can regulate the homeostasis of the auditory cortex to help refine the neural network (Teichert et al., 2017). Therefore, considering synaptic scaling in the regulation of synaptic pruning is becoming increasingly important.

After injury to the central nervous system, the brain must reorganize and optimize its neural networks to establish more effective neural circuits. Synaptic pruning plays a crucial role in this process. During normal development, synaptic pruning optimizes neural networks by eliminating redundant or ineffective synaptic connections, with microglia acting as the primary executors of this process (Paolicelli et al., 2011). Research has shown that at different developmental stages, rats exhibit synaptic reorganization capabilities following neuronal injury (Cotman et al., 1973). This age-dependent regulation of synaptic numbers highlights the dynamic nature of synaptic plasticity (Cotman et al., 1973). To elucidate whether synaptic pruning participates in the repair of injuries to the central nervous system, researchers established a T12 hemi-section model in mice and monitored changes in the dendritic spine numbers of pyramidal neurons in the hindlimb motor cortex *in vivo*. These authors reported that spine elimination peaked two weeks after spinal cord hemisection but was associated with the condition of normal mice after just one month. Synaptic remodeling occurs in the cortex contralateral to the injury site, suggesting the involvement of synaptic pruning in the early stages, with pruning effects diminishing during the recovery period (Zhang et al., 2021a). In another model of spinal cord injury, C–X3–C motif chemokine receptor 1 (CX3CR1) was found to mediate microglial pruning of the dendritic spines (Freria et al., 2017). Similarly, studies on peripheral nerve injury have indicated that these dynamic changes in synaptic density directly reflect the processes of synaptic pruning and remodeling. Campos et al. (2023) reported that the increased phagocytic activity of microglia was accompanied by a reduction in synaptic protein expression in a model of sciatic nerve injury; however, direct evidence for microglia-mediated synaptic pruning in this model, acquired from labeled synaptic proteins within microglia, was not reported. Research on peripheral nerve injuries has shown that microglia are involved in synaptic pruning, with the process being more dependent on recruitment following the activation of chemokine receptor 2 (Rotterman et al., 2019). Therefore, synaptic pruning plays a key role in regulating the neural networks of the central nervous system to cope with various neural injuries. This process involves not only the participation of microglia but also the regulatory mechanisms of neurons, as well as the influence of other cellular components and noncellular elements.

In this review, we focus on the important molecular mechanisms that trigger and regulate synaptic pruning, including complement molecules and chemokine signals, which initiate pruning through direct or indirect effects on glia‒neuron communication. Simultaneously, we review the latest research progress on the role of synaptic pruning in neurological diseases. Finally, we summarize the mechanisms and pathways by which synaptic pruning can influence diseases of the central nervous system and discuss the latest technologies and prospects in the field of synaptic research. Our aim was to provide comprehensive reference guidelines for researchers in this field.

## Search Strategy

**Figure 1:**
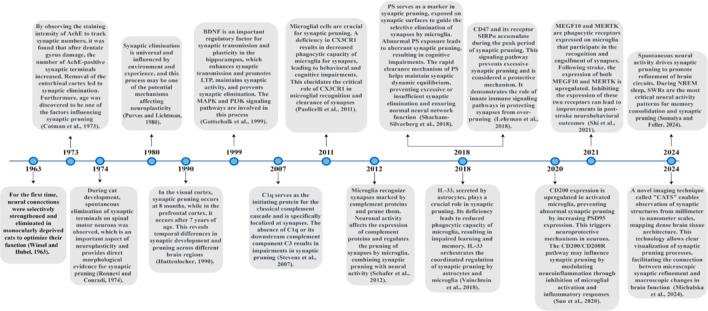
Timeline showing the historical evolution and research progress on the molecular mechanisms of synaptic pruning. Created with draw.io (www.drawio.com). AchE: Acetylcholinesterase; BDNF: brain-derived neurotrophic factor; C1q: component 1q; C3: component 3; CATS: comprehensive analysis of tissues across scales; CD200: cluster of differentiation 200; CD47: cluster of differentiation 47; CX3CR1: C-X3-C motif chemokine receptor 1; IL-33: interleukin 33; LTP: long-term potentiation; MAPK: mitogen-activated protein kinases; MEGF10: multiple EGF-like domains 10; MERTK: MER receptor tyrosine kinase; NREM: non-rapid eye movement; PI3K: phosphatidylinositol 3-kinase; PS: phospholipids; PSD95: postsynaptic density protein-95; SIRPα: signal regulatory protein α; SWRs: sharp wave ripples.

In this narrative review, we conducted a literature search in the PubMed database using the following keywords: microglia, synaptic pruning, synaptic plasticity, synapse elimination, neurodevelopment, neural circuit refinement, chemokines, and neurodegenerative diseases. To capture the historical evolution and latest advances in this field, our search covered literature from the 1960s, when the concept of synaptic pruning was first proposed, to 2024. While the phenomenon of synaptic pruning was discovered in the last century, the past decade has seen rapid development in the in-depth study of the molecular mechanisms underlying synaptic pruning and cutting-edge techniques. We have compiled a chronological account of the evolution of synaptic pruning research, highlighting major discoveries, which are visually represented in a timeline (**Figure 1**).

## Mechanisms Responsible for Synaptic Pruning

### Triggers of synaptic pruning

Neuronal plasticity refers to the ability of neurons to respond to various environmental stimuli and change their synaptic strength, morphology and functionality (Qi et al., 2024; Abokyi and Tse, 2025). Pruning the synapses and axons of damaged neurons can promote partial recovery of intact neural circuit functions (Singh and Donlea, 2020), thereby contributing to the maintenance of normal synaptic plasticity. Excessive activation of synaptic pruning in neurological diseases leads to synaptic loss, thus exacerbating the disease (Hong et al., 2016). This pruning process largely depends on the neuronal response to internal synaptic pruning commands and external stimuli. However, the pruning mechanism can be influenced by various factors, including neuronal activity patterns, neurotransmitter release and re-uptake, and cytokine effects. Consequently, the pruning processes triggered by different mechanisms can lead to alterations in synaptic connections and strength, which in turn impact neurodevelopment, learning and memory functions. Hereafter, we separately describe the mechanisms that can trigger synaptic pruning driven by spontaneous neural activity and experience-dependent neural activity, providing insights into the impact of synaptic pruning on brain function.

### Spontaneous synaptic pruning delivering by neural activity

Sensory experiences can guide the formation and consolidation of neuronal synaptic connections, whereas spontaneous activity is defined as the electrical activity generated by neurons and synapses in the absence of external stimulation. These two processes are interdependent and collaboratively promote the normal development of sensory circuits. Spontaneous activity facilitates the remodeling and elimination of synaptic connections between neurons, thus resulting in enhanced synaptic input that allows neurons to assess the overall environment comprehensively (Ronnevi and Conradi, 1974; Tononi and Cirelli, 2014). The mechanisms responsible for synaptic pruning driven by spontaneous neural activity are key factors in the normal development and functional maturation of sensory circuits and are crucial for appropriate synaptic development and the refinement of neural circuits (Assali et al., 2014; Kerschensteiner, 2014; Pumo et al., 2022; Somaiya and Feller, 2024). Furthermore, spontaneous neuronal activity can drive microglial contact with neurons (Hristovska et al., 2022). These findings provide significant insights into the mechanisms underlying synaptic pruning.

### Critical role of AMPAR-mediated spontaneous neural activity in synaptic refinement

The visual cortex serves as one of the most effective model systems for investigating synaptic pruning (Wiesel and Hubel, 1963; Huttenlocher, 1990). Within the visual system, the frequency of retinal waves is crucial for synaptic refinement in normal visual circuits (Burbridge et al., 2014). Spontaneous neuronal activity is a key component in the refinement of visual synaptic connections (Kirkby et al., 2013). AMPAR is a critical regulator of this spontaneous activity. In a previous experiment evaluating the impact of spontaneous activity on synaptic development in the mouse retina, researchers used tetrodotoxin to inhibit spontaneous activity and observed a reduced AMPAR amplitude and reduced synaptic refinement. In contrast, normal spontaneous activity drives the refinement and plasticity of retinal synapses (Hooks and Chen, 2006). Further studies on chronic cerebral hypoperfusion demonstrated that reduced AMPAR levels led to an increase in silent synapses and reduced synaptic refinement (Wang et al., 2016). AMPAR is central to mediating fast excitatory synaptic transmission. When synapses contain only NMDARs but lack AMPARs, synaptic silencing occurs (Liao et al., 1995). In early developmental hippocampal neurons, silent synapses typically contain only NMDARs and lack AMPARs (Durand et al., 1996). When AMPAR is blocked using the AMPAR antagonist CNQX, synaptic transmission is weakened or even completely blocked, and synapses quickly become silent synapses. However, through high-frequency, stimulation-induced and long-term potentiation (LTP), AMPARs are reinserted into the synaptic membrane, thereby restoring synaptic transmission function. Furthermore, in the mature hippocampal CA1 region, studies have shown that miR-34a is an important gene that regulates synapses in the mature brain. The deletion of this gene results in many silent synapses characterized by reduced AMPAR expression and increased dendritic spine density (Min et al., 2023). The literature indicates that sphingosine-1-phosphate increases the rate of AMPAR-mediated miniature excitatory postsynaptic currents (Kanno et al., 2010); however, by using calcium imaging to label spontaneous activity in neurons, we found that sphingosine-1-phosphate treatment significantly inhibited neuronal activity (Skoug et al., 2022). Interestingly, S1PR is located post-synaptically (Hajipour et al., 2023) and can be used to evaluate changes in spontaneous neuronal activity and elucidate its effects on synaptic elimination. In Fragile X syndrome, spontaneous neuronal activity is inhibited due to the significant loss of AMPAR in synapses and reduced EPSC amplitude, leading to impaired spontaneous synaptic transmission, which manifests as excessive dendritic branching and numerous spines (Kim and Cho, 2014). The activity of postsynaptic AMPARs is crucial for spontaneous neuronal activity and indirectly affects synaptic pruning and neural circuit composition.

### Transcription factors and associated molecules in the regulation of spontaneous neural activity and synaptic pruning

Transcription factor activity is an important factor in spontaneous activity (Pumo et al., 2022). Nuclear receptor subfamily 2 group F member 1 (Nr2f1) is a key transcription factor that encodes a member of the steroid hormone receptor superfamily during cortical development and is an upstream molecule that regulates spontaneous neuronal activity. The loss of Nr2f1 leads to reduced dendritic complexity, further resulting in reduced mitochondrial content; in addition, research has shown that dendritic spine density also decreases drastically, a phenomenon caused by mitochondrial dysfunction (Bonzano et al., 2023). One pertinent question is whether Nr2f1 can influence synaptic pruning through mitochondrial function, thereby leading to reduced dendritic spine density. A previous study by Baranov et al. (2021) showed that after mitochondrial membrane integrity is compromised, mitochondrial DNA is released into the cytoplasm, triggering a series of reactions that activate microglial phagocytosis in synapses. Therefore, although Nr2f1 deficiency does not directly reduce the number of dendritic spines, it can drive the microglial phagocytosis of synapses via mitochondrial pathways. Notably, Nr2f1 deficiency results in a significant reduction in hyperpolarization-activated cyclic nucleotide-gated cation 1 (HCN1) protein levels (Del Pino et al., 2020). HCN1 belongs to the voltage-gated sodium channel superfamily, is widely expressed in the cortex and hippocampus, and plays an important role in regulating neuronal activity and synaptic transmission (Combe and Gasparini, 2021). HCN1 drives spontaneous firing, mediating spontaneous neuronal activity and further promoting the synaptic pruning process (Yin et al., 2018). G protein-coupled estrogen receptor, a membrane-bound receptor, promotes HCN1 expression in the CA1 region; treatment with an agonist (G1) reduces the number of spine synapses while increasing the number of mature dendritic spines in the hippocampal neurons of female mice. Additionally, the lack of presynaptic companion proteins can influence synapse formation, leading to this contradictory phenomenon (Li et al., 2021b). Interaction between neurexin, a presynaptic companion protein, and neuroglin, a postsynaptic companion protein, can prevent glutamate-induced synaptic loss, whereas neuroligin deficiency leads to increased synaptic elimination (Sell et al., 2024). Furthermore, the absence of another class of synaptic companion protein, LRRTM1, results in reduced synaptic numbers in the medial prefrontal cortex (mPFC) region. Following LRRTM1 knockout, the expression of SynCAM1 increases significantly. This interdependent action jointly maintains the stability of synaptic numbers in the mPFC region. Notably, when both LRRTM1 and SynCAM1 are absent, their deficiency leads to reduced neurexin expression and spontaneous neuronal activity (de Arce et al., 2023). These findings indicate that LRRTM1 and SynCAM1 jointly maintain normal neural activity and neural network stability. These findings suggest that transcription factors not only play crucial roles in regulating spontaneous neuronal activity but also further promote synaptic pruning by driving spontaneous neural activity, thereby maintaining the stability and function of neural circuits.

### Molecules of neural activity–driven synaptic pruning in sensory neural circuits

Synaptic pruning of mitral cell dendritic spines in the olfactory system requires spontaneous neural activity. When Kir2.1 is overexpressed in mitral cells, glutamatergic synapses form on dendrites and inhibit spontaneous neuronal activity, leading to the accumulation of glutamatergic synapses (Fujimoto et al., 2023). Notably, Kir2.1 can induce microglia to release interleukin (IL)-1β, exacerbating inflammatory responses (Maejima et al., 2022). These findings suggest that interfering with Kir2.1 could activate spontaneous neuronal activity to promote synaptic pruning, with glutamatergic synapses as the likely target, and further reduce inflammatory responses, thereby protecting normal synaptic connections and refinement in the nervous system. Otof is known to be a major gene involved in sensory system development. Following the knockout of Otof, hearing is lost, and synaptic elimination is enhanced in the auditory circuit from the medial nucleus of the trapezoid body (MNTB) to the lateral superior olive (LSO), with extensive MNTB-LSO inputs leading to a further reduction in synaptic refinement (Müller et al., 2019). Changes in spontaneous synaptic membrane potential induce synaptic activity. Gene intervention, which disrupts spontaneous retinal activity, results in significant reductions in retinal synaptic density and impaired synaptic refinement (Zhang et al., 2023a). These results indicate that spontaneous neuronal activity plays a crucial role in synaptic pruning across multiple sensory systems; consequently, intervening with relevant genes or molecular pathways may become an important approach for protecting synaptic connections and functions in the nervous system.

To summarize, the neural activity regulated by specific ion channels or molecules plays a crucial role in synaptic pruning across multiple sensory systems. Targeting these different classes of signaling molecules by molecular intervention may represent an important direction for protecting synaptic connections and maintaining normal neural functionality in the nervous system.

### Experience-dependent synaptic pruning

Sensory experience refers to the ability of an organism to adapt to a constantly changing sensory environment, a capacity that relies on the ability of the nervous system to self-regulate external stimuli. Sensory deprivation experiments (e.g., visual, auditory, and olfactory deprivation) have revealed how the brain relies on experience to modulate synaptic function. Initially, Wiesel et al. demonstrated that monocular visual deprivation in cats led to corresponding deficits in neural connectivity. However, the provision of visual experience one or two months prior to deprivation alleviated these defects, and adult cats showed stronger adaptive responses to visual deprivation (Wiesel and Hubel, 1963). These early findings from sensory deprivation experiments showed that experience-dependent plasticity could manipulate neuronal activity. Moreover, such experiences optimize neural circuits via mechanisms such as LTP and long-term depression (LTD) to respond to sensory input, thereby maintaining synaptic plasticity homeostasis. Key mechanisms for the suppression of neuronal activity in neurons of the visual cortex following monocular deprivation include the sliding threshold model and synaptic scaling to regulate neuronal activity, maintaining the excitation/inhibition balance, as well as cross-modal plasticity compensating for sensory loss via Hebbian mechanisms (Whitt et al., 2014). Therefore, an imbalance in the mechanisms that maintain neural network stability may lead to severe disorders in neural development and function.

### Experience-dependent synaptic pruning mediated by long-term depression and long-term potentiation

Numerous studies employing visual deprivation, auditory deprivation, enriched environments, and motor learning tasks have elucidated how long-term influences from both the internal and external environments induce experience-dependent changes in neuronal connectivity (Rodriguez et al., 2019; Campelo et al., 2020; Faust et al., 2021; He et al., 2021; Nishibe et al., 2022). The size, morphology and number of dendritic spines undergo modifications throughout brain developmental stages, indicating the formation and elimination of synapses, which constitute the basis of experience-dependent synaptic plasticity (Hill and Zito, 2013; Stein and Zito, 2019). Research has demonstrated that LTP increases dendritic spine density, while LTD induces synaptic pruning (Chidambaram et al., 2019). Under experience-driven conditions, prolonged LTD results in the gradual elimination of persistently inefficient synapses (Tanaka et al., 2020). The primary forms of LTD-induced synaptic pruning are NMDAR dependent, mGluR-dependent and heterosynaptic. NMDAR activation is crucial for LTD and represents one of the most significant forms of synaptic plasticity (Sawchuk et al., 2020; Shin et al., 2020; Thomazeau et al., 2021). Sidorov et al. (2015) investigated slices of the visual cortex from monocularly deprived mice and reported that NMDAR-dependent LTD mechanisms facilitated experience-dependent synaptic structural plasticity. Furthermore, these mechanisms are regulated by postsynaptic Ca^2+^ concentrations and contribute to hippocampal synaptic pruning (Wiegert and Oertner, 2013). Synaptic plasticity and experience-dependent refinement of neural circuits constitute fundamental processes in neurodevelopment. Recent findings have highlighted the intricate interplay between neurotrophin signaling, calcium dynamics and activity-dependent synaptic remodeling in the developing visual cortex. p75NTR, which is predominantly localized in dendritic spines, plays a crucial role in synaptic plasticity, with its absence impairing NMDA-dependent LTD (Woo et al., 2005). p75 is a low-affinity receptor for brain-derived neurotrophic factor (BDNF), while tyrosine receptor kinase B (TrkB) is a high-affinity receptor. BDNF-dependent LTP is associated primarily with TrkB activation; in contrast, the activation of p75NTR is linked to LTD and promotes spine elimination (Wiegert and Oertner, 2013). This complex interaction contributes to the fine-tuning of synaptic strength and structure, necessitating the expression of BDNF (Aarse et al., 2016). Recent studies indicated that the precursor form of BDNF, proBDNF, preferentially binds to p75NTR and induces excitatory synaptic loss following oxygen and glucose deprivation (OGD). In this context, activated microglia synthesize BDNF, thus accelerating synaptic loss, while miniature excitatory postsynaptic current amplitude is suppressed (Cramer et al., 2022). This synaptic loss may be mediated by microglia-driven synaptic pruning. Furthermore, neurotrophic factors may also play a role in LTD dependent, experience-driven synaptic pruning. The mechanisms of LTD-induced synaptic pruning involve various other genes, such as activity-regulated cytoskeleton-associated protein (Arc) and myocyte enhancer factor 2 (MEF2); MEF2 induces LTD and further eliminates synapses by activating Arc (Wilkerson et al., 2014; Chang et al., 2017). BDNF, a downstream transcription factor of MEF2, is known to be regulated by MEF2 (Avarlaid et al., 2024). BDNF also enhances the expression of Arc in the dendrites of hippocampal neurons (Baldinotti et al., 2025). Thus, neurotrophic factors trigger synaptic pruning by inducing LTD, and other molecular mechanisms can reciprocally influence the expression and functionality of BDNF. This intricate interaction plays a role in the regulation, remodeling and refinement of neural networks.

BDNF can promote synaptic transmission and regulate synaptic plasticity (Gottschalk et al., 1999); the release of BDNF is essential for experience-dependent visual cortex refinement. Tanaka et al. (2020) reported that the triggering of LTD when the postsynaptic calcium concentration was moderately increased subsequently promoted the release of proBDNF and that this proBDNF bound to p75NTR to recognize and eliminate null synapses, thus proving that LTD is critical for experience-dependent synaptic pruning. While p75NTR is crucial for maintaining dynamic changes in dendritic spine density, its non-specific binding to various neurotrophic factors indicates that identifying specific endogenous ligands, such as proBDNF, could further delineate its role in regulating synaptic pruning and NMDA-dependent LTD via Ca^2+^ signaling. Wang et al. (2022b) demonstrated that NMDARs promote the release of BDNF by increasing presynaptic calcium content. BDNF, translated into proBDNF, then binds to p75NTR to promote LTD, thereby triggering synaptic pruning. This Ca^2+^-dependent mechanism is one of the necessary mechanisms for LTD to regulate synaptic pruning. Additionally, glucocorticoids increase under stress, and glucocorticoid receptors inhibit the activation of TrkB and the production of LTP. This is one factor that can influence LTD and LTP. Another factor is the level of NMDAR activation; moderate activation of NMDARs leads to an increase in postsynaptic Ca^2+^ content, thereby triggering LTD, while stronger activation leads to a further increase in Ca^2+^, thereby triggering LTP (Peters et al., 2018). Therefore, NMDARs can regulate synaptic pruning via a Ca^2+^-dependent mechanism, and both BDNF and p75NTR should be considered while also taking into account the intensity of LTD and LTP when assessing synaptic pruning. In the primary visual cortex, neurogranin (Ng) also facilitates experience-dependent synaptic pruning, which is essential for the functional optimization of neural circuits. Ng plays a crucial role in maintaining the equilibrium of AMPAR-mediated synaptic transmission during the critical period in the mouse primary visual cortex. Under normal visual conditions, Ng deficiency results in reduced AMPAR-positive synapse numbers, impaired maturation of AMPAR-silent synapses, and increased spine elimination. Visual deprivation prevented the synapse loss caused by Ng deficiency. This phenomenon can be attributed to Ng deficiency augmenting LTD induction in layer 2/3 pyramidal neurons in V1, thus facilitating the elimination of AMPAR-positive synapses and impeding the maturation of silent synapses. Sensory experience modulates the ability of Ng to coordinate this process through the Ca^2+^/CaM pathway, thereby refining neural circuits (Han et al., 2017). In conclusion, LTD- and LTP-mediated experience-dependent synaptic pruning play pivotal roles in neural circuit optimization and functional plasticity. Elucidating the molecular mechanisms underlying these processes is essential if we are to advance our understanding of neurodevelopment and formulate novel therapeutic strategies for neurological disorders.

### Glutamate A3-mediated experience-dependent synaptic pruning in the auditory pathway

In the auditory pathway, auditory experience induces plastic changes in the mechanisms by which auditory information is processed within the auditory system. The presence of glutamate A3 (GluA3) at the axonal terminals of auditory neurons is essential for the transmission and processing of auditory information. In GluA3-knockout mice, a reduction in AN-BC synapses was observed, thus resulting in hearing loss. To eliminate aging-related factors, an auditory signal processing system identified reduced ABR amplitudes on postnatal days 2 and 3 due to diminished AN activity. The underlying glutamatergic mechanism involves abnormal AMPAR subunit assembly following GluA3 deletion, thus preventing effective synaptic transmission and causing functional synaptic reduction (García-Hernández et al., 2017). Another study demonstrated that in synaptic transmission at AN-BC synapses, the absence of GluA3 led to a reduced number of AMPARs and a delayed response in EPSCs (Antunes et al., 2020). Following sensory deprivation in the ventral posteromedial nucleus, a reduction in the amplitude of AMPAR-EPSCs was observed. However, there is no direct evidence that sensory experiences selectively enhance the trafficking of GluA3-containing AMPARs (Wang et al., 2011). Several hypotheses may explain this phenomenon: (1) *in vitro* patch-clamp detection of the membrane potential in tissue slices may not accurately represent *in vivo* changes; (2) signals from NMDARs and some AMPAR receptors potentially initiate synaptic elimination programs; (3) variations in glutamatergic mechanisms involved in sensory pathways across brain regions or the potential involvement of other GluA subunits of AMPAR in the synaptic pruning process; and (4) the experience-dependent upregulation of AMPAR may depend on the presence of GluA3, a mechanism that is especially critical within the auditory system. Consequently, the presence of GluA3 is not only crucial for the normal function of the auditory system but also modulates the dynamic assembly of AMPAR subunits via experience-dependent mechanisms; this is fundamental for maintaining neuroplasticity in the auditory pathway. GluA3 is a critical molecule in the transmission of auditory information, and early auditory experiences are similarly important in maintaining the homeostasis of auditory neural networks.

## “Eat-Me” Signaling Pathways

### Complementary signal

**Figure 2:**
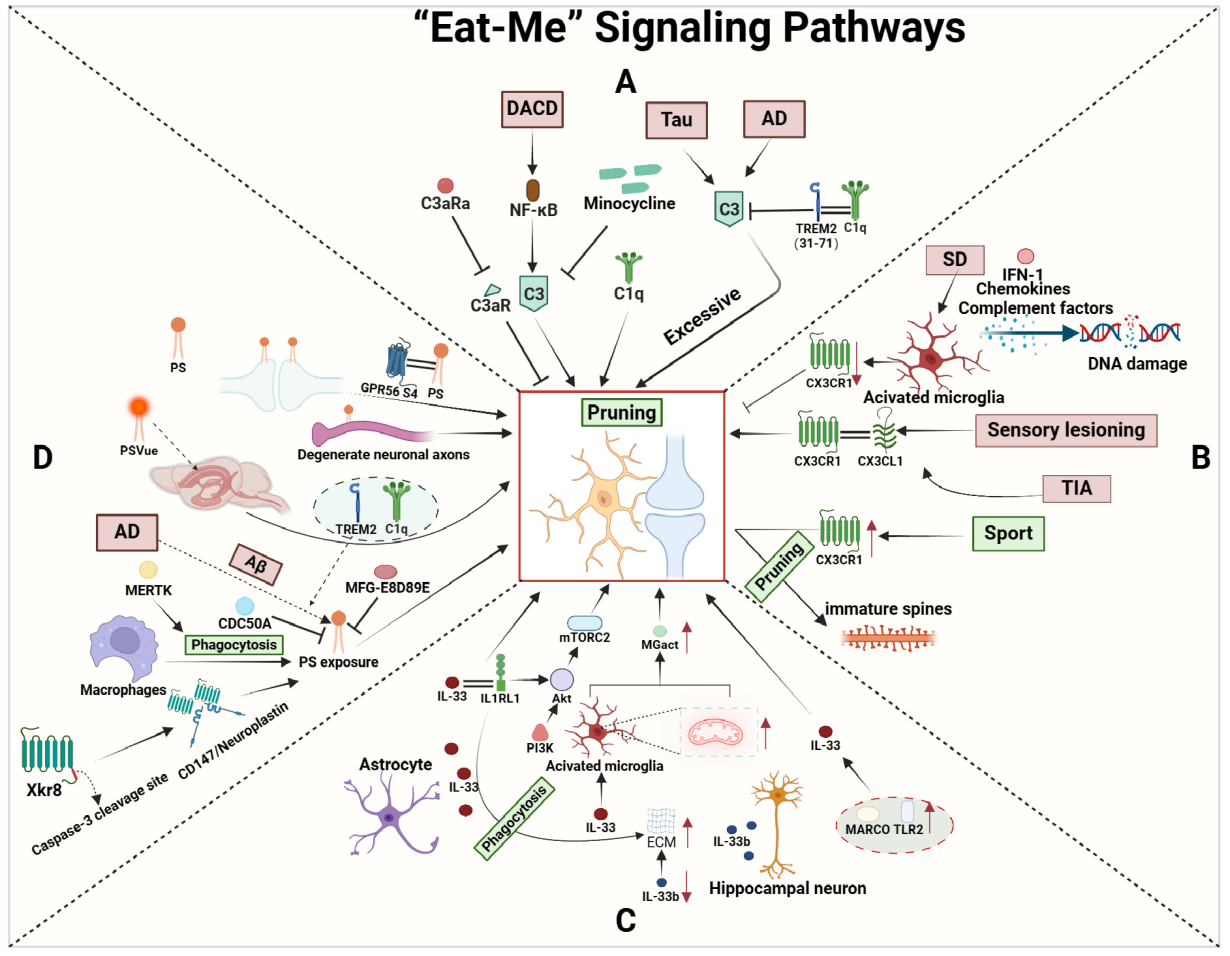
“Eat-Me” signaling pathways in synaptic pruning. (A) Role of complement signaling in synaptic pruning: Microglia-derived C1q mediates recognition and pruning of C1q-tagged synapses. C3aRa inhibits synaptic pruning by blocking C3aR. NF-κB promotes C3-mediated synaptic pruning, a process inhibited by minocycline. TRME2 and C1q binding can inhibit C3. (B) Role of CX3CL1/CX3CR1 signaling in synaptic pruning: CX3CR1 binding to CX3CL1 mediates microglial synaptic pruning. CX3CR1 and CX3CL1 binding promote synaptic pruning. Sleep deprivation leads to microglial overactivation, causing CX3CR1 downregulation and impaired synaptic pruning. Exercise upregulates CX3CR1 expression, promoting the pruning of immature dendritic spines by microglia. (C) IL-33/IL1RL1 signaling in synaptic pruning: Astrocyte-derived IL-33 binds to microglial IL1RL1, mediating microglial synaptic pruning. This binding initiates PI3K/AKt/mTORC2 transcriptional responses in microglia. IL-33 enhances microglial mitochondrial activity and MGact phagocytic gene expression. Upregulation of MARCO and TLR2 expression promotes IL-33 expression. Knockout of hippocampal neuron-expressed IL-33b leads to ECM accumulation around synapses. (D) Phosphatidylserine signaling in synaptic pruning: Xkr8 forms a tetramer with CD147 or neuroplastin, causing PS exposure on the cell surface for macrophage recognition and phagocytosis. PS exposure of degenerating axons targets them for microglial phagocytosis. Synaptic PS exposure mediates synaptic pruning, a process inhibited by MFG-E8D89E. GPR56 S4 binding to PS promotes microglial synaptic phagocytosis. CDC50A inhibits PS exposure, whereas MERTK promotes microglial synaptic phagocytosis. PSVue injection into the lateral ventricle can promote synaptic pruning. Created with BioRender.com. AD: Alzheimer’s disease; Akt: protein kinase B; C1q: component 1q; C3: component 3; C3aR: complement c3a receptor; C3aRa: complement C3 receptor antagonist; CD147: cluster of differentiation 147; CDC50A: cell cycle control protein 50A; CX3CL1: C-X3-C motif chemokine ligand 1; CX3CR1: C-X3-C motif chemokine receptor 1; DACD: Diabetes-associated cognitive dysfunction; ECM: extracellular matrix; GPR56: G protein-coupled receptor 56; IFN-1: interferon 1; IL1RL1: interleukin-1 receptor-like 1; IL-33: interleukin 33; MARCO: macrophage receptor with collagenous structure; MERTK: mer tyrosine kinase; mTORC2: mechanistic target of rapamycin 2; NF-κB: nuclear factor kappa-B; PI3K: phosphatidylinositol-3-kinase; PS: phosphatidylserine; SD: sleep deprivation; TIA: transient ischemic attack; TLR2: toll like receptor 2; TREM2: triggering receptor expressed on myeloid cells-2; Xkr8: XK-associated protein 8.

The complement cascade, a critical component of the innate immune surveillance system, is essential for synaptic pruning in the nervous system. Complement 1 (C1), specifically C1q, is known to initiate the classical complement pathway and, together with downstream complement protein C3, facilitates synaptic pruning (Stevens et al., 2007). A substantial body of literature demonstrates that activation of the complement pathway induces the elimination of inefficient synaptic connections and refines neural circuits (Schartz and Tenner, 2020; Wang et al., 2020; Gomez-Arboledas et al., 2021). Under pathological conditions, complement proteins, primarily C1q, mark excess and unnecessary synaptic connections. These marked synapses are then recognized by receptors on the microglia and are phagocytosed, thus facilitating synaptic refinement. For example, in AD, microglial-derived C1q colocalization with postsynaptic density protein 95 (PSD95) intensifies with the accumulation of amyloid-β (Aβ) plaques, thus resulting in synaptic loss and neuronal dysfunction (Hong et al., 2016). In sepsis-associated encephalopathy (SAE), C1q-tagged synapses are catabolized in microglial lysosomes, and inhibiting C1q activation or reducing the number of microglia can attenuate synaptic loss (Chung et al., 2023). In an experiment aimed at alleviating visceral pain, environmental enrichment was found to mitigate microglial synaptic pruning by reducing the expression levels of C1q and CR3, rather than C3, in the central nucleus of the amygdala, demonstrating the efficacious alleviation of visceral pain. These findings suggest that the complement cascade is a crucial mechanism for microglial synaptic pruning (Yuan et al., 2022). In the developing dorsal lateral geniculate nucleus (dLGN), the expression of C3 reaches its maximum postnatally but decreases by postnatal day 9 (P9). Pharmacological inhibition of microglia and knockout of C3 results in diminished microglial phagocytic capacity and elevated synaptic numbers, causing defects in dLGN neural circuit integrity (Schafer et al., 2012). Conversely, in models of AD, C3 gene deficiency can mitigate excessive synaptic pruning. Furthermore, the absence of C3 and its high-affinity receptor CR3 on macrophages can alleviate Aβ-induced synaptic loss (Chung et al., 2023). In brains afflicted with tau pathology, the expression level of C3 is elevated, leading to synaptic loss triggered by the activation of the complement cascade. The absence of C3 can significantly rescue the reduction in dendritic spine density, providing neuroprotection in models of tau pathology (Wu et al., 2019). Research indicates that the binding of triggering receptor expressed on myeloid cells-2 (TREM2) peptide segments 31–71 to C1q suppresses the expression of C3 and alleviates the excessive microglial synaptic pruning caused by complement activation (Zhong et al., 2023). Consequently, the application of TREM2-targeted interventions represents an effective method for mitigating excessive microglial synaptic pruning mediated by complement cascade activation in the context of AD. In a mouse model of diabetes-related cognitive impairment, abnormal activation of nuclear factor kappa-B (NF-κB) upregulated the expression of C3, thus enhancing synaptic pruning. However, the inhibition of C3aR in microglia was shown to attenuate synaptic loss and reinstate synaptic plasticity (Jiang et al., 2023). These findings identified complement factors as crucial mediators of microglial synaptic pruning. However, further research is imperative to delineate the specific mechanisms of the complement system; such research will enhance our understanding of the complex roles of the complement system in health and disease, and potentially guiding therapeutic strategies (**Figure 2**).

### CX3CL1/CX3CR1 signaling

Chemokines are a family of small-molecule cytokine proteins that interact with seven-transmembrane G protein-coupled receptors (GPCRs). Upon receptor engagement, chemokines facilitate cell migration to specific regions under both homeostatic and inflammatory conditions. This directional migration is mediated by intracellular signaling pathways initiated by receptor activation. Chemokine receptors are differentially expressed on target cell surfaces and are classified according to the chemokine family they bind, such as XCR, CCR, CXCR, and CX3CR. Significantly, C–X3–C motif chemokine ligand 1 (CX3CL1), also known as fractalkine, is predominantly expressed by neurons and is implicated in key biological processes, including neuromodulation, neurotransmission and neurogenesis (Angelopoulou et al., 2020). The specific receptor of CX3CL1, CX3CR1, is abundantly expressed in monocyte-macrophages and microglia. CX3CR1 functions as a specific promoter for microglia and modulates their communication with neurons (Camacho-Hernández and Peña-Ortega, 2023). Furthermore, CX3CR1 is crucial for regulating the morphodynamic changes of microglia during their interactions with neurons (Hristovska et al., 2022). In CX3CR1-deficient mice, there is a reduction in microglial numbers and a retardation in synaptic pruning, ultimately resulting in an excess of immature synapses that impair neural transmission efficiency (Paolicelli et al., 2011). Consequently, understanding the molecular mechanisms by which microglia prune synapses will increase our understanding of the processes used to refine neural circuits. Sleep deprivation induces the downregulation of CX3CR1 expression in the hippocampus of both adolescent and adult mice. However, only in adolescent mice does this lead to the generation of excessive and weakly active synapses. Further investigation revealed that this phenomenon is due to the impaired phagocytic function of microglia during adolescence, which disrupts the synaptic pruning process (Tuan and Lee, 2019). A separate study demonstrated that following sleep deprivation in adolescent mice, CX3CR1 expression in the hippocampus was also downregulated. Interestingly, enhanced microglial phagocytic function was observed exclusively in the dentate gyrus and CA1 regions, while microglia in the CA3 region remained inactive (Wang et al., 2023). Similarly, Sahasrabuddhe et al. induced microglial activation in neonatal mice by administering CX3CR1-Cre. Transcriptomic analysis revealed the upregulation of interferon (IFN) pathways, the complement system, the major histocompatibility complex, and chemokine signaling pathways, thus resulting in excessive synaptic pruning by microglia (Sahasrabuddhe and Ghosh, 2022). Collectively, these findings suggest that multiple signaling networks, including the chemokine signaling pathway represented by CX3CR1 and the complement system, regulate microglia-mediated synaptic pruning in a collaborative manner. Moreover, this process appears to be not only age-dependent but also likely brain region specific. However, sustained exercise has been shown to increase CX3CR1 expression, thus facilitating the microglial pruning of immature dendritic spines and thus reinstating normal spine morphology (Tuan et al., 2021). The activation of CX3CR1-Cre by tamoxifen induced abnormal early postnatal microglial activation, culminating in excessive synaptic pruning associated with anxiety-like behavior in mice but without adverse effects on adult microglia. Transcriptome analysis further revealed significant upregulation of IFN-1, chemokine, and complement factor signaling in abnormally activated microglia. This induction results in DNA damage, further impacting the cell cycle and resulting in reduced proliferation and cell death (Sahasrabuddhe and Ghosh, 2022). Collectively, these findings demonstrated that CX3CR1 modulates microglial function in synaptic pruning and elimination to preserve homeostasis and refine neural circuits.

Numerous studies have demonstrated the crucial role of the CX3CL1/CX3CR1 axis in synaptic development and pruning. Gunner et al. (2019) demonstrated that sensory injury induced microglia-mediated synaptic pruning, a process dependent on the cooperative action of the CX3CL1/CX3CR1 axis. Similarly, Song et al. (2022) reported that this axis is essential for the development and maturation of ribbon synapses in the mouse cochlea. These authors reported high expression levels of CX3CR1 within cochlear macrophages on day 7, but following the administration of AZD8797 (an inhibitor of CX3CR1) by intraperitoneal injection for 7 consecutive days, the expression level decreased, and synaptic morphology and number abnormalities were observed, thus affecting hearing. Exogenous CX3CL1 administration increased CX3CR1 expression in macrophages, facilitating synaptic pruning and reinstating hearing. *In vitro* experiments also revealed that CX3CL1 can stimulate CX3CR1 expression in microglia and promote their phagocytic pruning of synapses (Wang et al., 2023). These data prove that this axis is integral for the establishment and maintenance of neural circuits. However, not all regulation of synaptic refinement is affected by the CX3CL1/CX3CR1 axis. For example, CX3CR1 knockout did not result in disordered synaptic pruning in cerebellar climbing fibers (Kaiser et al., 2020). Furthermore, Zheng et al. (2022) demonstrated that following transient ischemic attack (TIA), Annexin A1 (ANXA1) enhances the CX3CL1-CX3CR1 interaction, leading to excessive synaptic pruning. Inhibition of CX3CL1/CX3CR1 axis overactivation mitigated this effect, improving learning and memory in a mouse model of TIA. In a harmaline-induced rat model of epilepsy, microglial activation and CX3CR1 expression were significantly increased and modulated via the NF-κB/TLR4 pathway (Wu et al., 2023). These studies highlight the critical role of the CX3CL1/CX3CR1 axis in neural development, synaptic elimination and plasticity.

In conclusion, research on the role of chemokines in synaptic pruning not only elucidates the regulatory mechanisms of neuronal development but also highlights the complex processes of neural network formation. The CX3CL1/CX3CR1 axis, in particular, exerts a pivotal influence on synaptic pruning and preserves the stability of neural circuits. However, microglia may exhibit diverse functions at various developmental stages, and the influence of this axis may not be universally applicable to all brain regions. Consequently, comprehensive research into the specific roles and regulatory mechanisms of the CX3CL1/CX3CR1 axis in different neurological diseases is crucial if we are to develop tailored therapeutic strategies (**Figure 2**).

### Interleukin-33/IL1RL1 signaling

The refinement of neural circuits depends on synaptic pruning, a process primarily executed by microglia. Communication between the immune system and nervous system is crucial for maintaining brain homeostasis and mediating the mechanisms underlying neurological diseases. In this context, IL-33 and its receptor IL1RL1 play key roles in regulating synaptic quantity and maturation (Delmas and Dalmas, 2018). IL-33, which is primarily secreted by astrocytes, has been shown to promote excitatory synapse formation and transmission in hippocampal neurons and to play a crucial role in maintaining synaptic plasticity homeostasis (Wang et al., 2021). Recent studies have revealed the important role of IL-33/IL1RL1 in synaptic pruning and elimination. Vainchtein et al. (2018) used whole-cell patch-clamp techniques to show that IL-33 knockout led to an increase in glutamatergic synapses in the ventrobasal nucleus, thus resulting in synaptic overexcitation. In conjunction with the RNA sequencing results, these authors demonstrated that IL-33 regulated synaptic pruning by acting on the IL1RL1 receptor on microglia. Furthermore, He et al. (2022) used flow cytometry sorting and single-cell sequencing to demonstrate that microglia with increased phagocytic capacity presented increased levels of mitochondrial activity. These authors reported that IL-33 was highly expressed in microglia in the hippocampal area. In vivo injection of exogenous IL-33 significantly induced microglial activation, enhanced mitochondrial activity, and upregulated the expression of the phagocytic gene MGact. These authors also demonstrated that IL-33/IL-1RL1 deficiency leads to microglial dysfunction and reduced synaptic pruning refinement, ultimately resulting in synaptic and behavioral abnormalities. Further research revealed the molecular mechanism by which IL-33 regulates microglial synaptic pruning; this process was shown to be dependent on the PI3K/mTORC2/Akt signaling pathway, identifying Akt as a core downstream molecule of IL-33/IL-1RL1. High-dose IL-33 administration accelerated synaptic depletion in the mouse thalamus. Transcriptome analysis demonstrated the upregulation of pattern recognition receptors (MARCO and TLR2), both of which increase the functionality of IL-33 s in the promotion of synaptic pruning, thus highlighting the potential role of pattern recognition receptors in the regulation of synaptic refinement (Han et al., 2023). Collectively, these studies demonstrated that immune signaling mediated by IL-33 and IL1RL1 is crucial for synaptic pruning and maintaining homeostasis in the central nervous system.

Unlike IL-33, which is expressed primarily in astrocytes, Nguyen et al. (2020) reported that IL-33b, a subtype of IL-33, is expressed predominantly in hippocampal neurons. By creating a conditional knockout of IL-33b, these authors reported a reduction in the number of dendritic spines, accompanied by the accumulation of ECM around synapses. This accumulation impaired synaptic plasticity and resulted in reduced learning and memory abilities in the mice. Further research revealed that the enhanced function of IL-33 regulates microglial phagocytosis of the ECM via its receptor IL1RL1, thereby promoting synaptic plasticity. These findings suggest that the clearance of excess ECM by microglia may facilitate their full contact with neurons and participation in synaptic pruning. However, in the absence of IL-33, microglial phagocytic function is impaired, thus resulting in an increase in the number of dendritic spines. These additional spines are immature and can cause adverse effects on the maintenance of synaptic plasticity (Carlock et al., 2017; Han et al., 2023). Therefore, there is significant complexity in gaining an accurate understanding of whether the mechanism by which IL-33 regulates microglial pruning of synapses in the brain is instructive or cell specific. A comprehensive investigation of the role of IL-33 in optimizing neural circuits is now essential if we are to gain new insights into the homeostasis of brain synaptic plasticity and cognitive repair (**Figure 2**).

### Phosphatidylserine signaling

Phospholipids are a class of phosphorus-containing lipid molecules that serve as the primary components of biological membranes. Among these phospholipids, PS is synthesized when glycerophospholipids undergo an esterification reaction between their phosphate group and serine. PS supplementation has been shown to significantly enhance learning and memory in the brain (Doma et al., 2023). During apoptosis, PS activity is disrupted by the cleavage of membrane XK-associated protein 8 (Xkr8) at the C-terminus by two caspases. This cleavage results in the formation of tetramers with type I membrane proteins, CD147 or neuroplastin, which embed in the plasma membrane, exposing PS extracellularly. Consequently, PS is recognized and phagocytosed by macrophages (Suzuki et al., 2016). Furthermore, when the axons of degenerative neurons are exposed to PS, they can target microglia, promoting neuron‒microglia contact and subsequent phagocytosis (Shacham-Silverberg et al., 2018; Almasieh et al., 2022). Recent studies injected the zinc-activated PS-binding probe PSVue into the lateral ventricles of early postnatal mice and demonstrated that synaptic pruning by microglia in the hippocampus and retinal ganglia cells (RGCs) occurs. These authors reported that in the absence of phagocytic receptors (TREM2 and C1q), microglial synaptic pruning was impaired (Scott-Hewitt et al., 2020). These findings suggest that PS is a crucial signal for microglia-mediated synaptic pruning. Furthermore, Li et al. (2020) reported that GPR56, a GPCR, is highly expressed in the dLGN of mice between postnatal days 3 and 6. GPR56 S4, a subtype of GPR56, is the principal molecule responsible for regulating microglial pruning in RGC synapses. Loss of GPR56 impaired the pruning of PS-marked synapses by microglia. Subsequent investigations revealed increased RGC synaptic density and reduced microglial phagocytosis in PS-labeled synapses during early development, as well as reduced synaptic refinement in the dLGN. These findings indicate that GPR56 S4 directly binds PS and regulates microglial synaptic pruning through PS (Li et al., 2023b).

Recent studies have investigated whether PS mediates microglial synaptic pruning in adult neurons. Kurematsu et al. (2022) reported that the pruning of adult neurons in the olfactory bulb and hippocampal dentate gyrus by microglia depends on PS exposure at synapses, preferentially targeting immature dendritic spines. This process was inhibited by the dominant-negative milk fat globule EGF factor 8 D89E (MFG-E8D89E), resulting in impaired synaptic pruning. CDC50A, a cofactor of phospholipid flippase, is known to prevent PS exposure (Li et al., 2023a). Research has shown that CDC50A expression peaks in the brains of 6-month-old mice and is positively correlated with synaptic density. The knockdown of CDC50A in primary cultured neurons via siRNA increased PS exposure at synapses, inducing microglial synapse elimination. Similarly, the knockout of CDC50A in the dLGN yielded results that were consistent with those of the *in vitro* experiments. In GPR56 conditional knockout mice with additional CDC50A deletion, the reduction in synaptic density was less pronounced than that in controls (Li et al., 2021a), suggesting that GPR56 and CDC50A jointly regulate synaptic pruning. Park et al. (2021) demonstrated that PS acts as an initiating signal for microglial pruning in inhibitory synapses. Furthermore, the deletion of CDC50A in mature neurons increased PS exposure, leading to inhibitory postsynaptic loss. Introduction of the microglial phagocytic receptor Mer tyrosine kinase (MERTK) further enhanced synaptic elimination, whereas knockout of MERTK reversed the loss of inhibitory postsynapses caused by CDC50A deletion. These findings indicate that MERTK is another crucial molecule in microglial synaptic pruning and could serve as a therapeutic target for neurological disorders caused by excitatory-inhibitory (E/I) imbalance.

Although PS exposure promotes the pruning of synapses to maintain normal functionality in neural circuits, this can also yield beneficial effects in disease states. For example, in AD, Aβ stimulation leads to dendritic spine hyperactivity and induces PS exposure on synapses, thus enabling microglia to phagocytose these abnormal synapses. Moreover, TREM2 serves as a critical molecule that activates microglial phagocytosis and regulates Aβ-induced PS tagging of abnormal synapses (Popescu et al., 2023). In neurons containing Tau fibrillary tangles, PS exposure similarly induces microglial phagocytosis of abnormal neurons to maintain normal neuronal functionality (Brelstaff et al., 2018). This process eliminates the disruptive effects of abnormal synapses on neural circuits, thus preserving normal signal transmission in neurons.

These findings emphasize the pivotal role of PS in the establishment and pruning of synaptic connections, highlighting its importance in promoting synaptic maturation and reinforcing the formation and strengthening of functional brain circuits. Furthermore, PS may serve as a biomarker for certain neurological disorders. Given its importance, PS could represent a promising therapeutic target for future treatments of neurodevelopmental disorders arising from synaptic pruning dysfunction (**Figure 2**).

In conclusion, during the “eat-me” signal transduction process of synaptic pruning, microglia exert their synaptic pruning function in a manner that is mediated by complement factors, chemokines, and other molecules, thereby eliminating abnormal synapses and restoring normal functionality in neural circuits. Astrocyte-derived IL-33 also plays a crucial role in this process. However, it is important to recognize that in many pathological conditions, this pruning is not necessarily beneficial. Identifying the core targets within these mechanisms that promote synaptic pruning and determining if they can serve as a “switch” to regulate this process remains a key focus for future neuroscience research.

## “Don’t eat me” Signaling Pathways

### CD47/signal regulatory protein α signaling

Cluster of differentiation 47 (CD47) is a transmembrane protein belonging to the immunoglobulin superfamily that is widely expressed on various cell surfaces. CD47 binds to signal regulatory protein α (SIRPα) on immune cells, leading to the activation of immunoreceptor tyrosine-based inhibition motifs within SIRPα. This interaction recruits the tyrosine phosphatases SHP-1/2, thereby preventing immune cells from phagocytosing self-cells (Lemke, 2019). In oncology, this function has been targeted as a strategy to inhibit tumor growth. Recently, research on CD47/SIRPα has gained prominence in the field of neuroscience. Both neurons and glial cells are known to express CD47, thus highlighting its critical role in neuroimmune regulation, particularly in maintaining microglial homeostasis during neuroinflammatory conditions (Gheibihayat et al., 2021). Moreover, accumulating evidence suggests that CD47/SIRPα plays a significant role in synaptic plasticity. For example, in a mouse model of cancer-induced pain, microglia were shown to participate in pruning GABAergic synapses in the spinal cord dorsal horn in a process that was associated with reduced CD47/SIRPα expression (Zhang et al., 2024b). When SIRPα was knocked out in the mouse brain, excessive pruning of RGC synapses by microglia occurred. Similarly, *in vitro* experiments revealed that SIRPα KO microglia effectively phagocytosed CD47 KO-labeled synaptosomes. These lines of evidence show that the CD47/SIRPα interaction can prevent excessive pruning of synapses by microglia, thereby preventing impaired synaptic refinement. Synaptic protection is a crucial mechanism during normal neural development. Lehrman et al. (2018) reported that CD47 was primarily localized at synapses within the postnatal dLGN of normal mice. Research has shown that SIRPα deficiency in microglia enhances their phagocytic activity. Importantly, the knockout of SIRPα impaired CD47 recognition, thus resulting in exacerbated synaptic pruning and cognitive deficits. Furthermore, these authors reported that tetrodotoxin-induced suppression of neuronal activity downregulated CD47 expression, rendering less active synapses more susceptible to elimination (Ding et al., 2021). These data suggest that CD47/SIRPα is a crucial factor in the regulation of microglial synaptic pruning. Age-related neural dysfunction has long been a focus of neuroscience research. DeVries et al. utilized the dorsolateral prefrontal cortex (dlPFC) of rhesus monkeys of different ages as a model to study changes in synaptic pruning associated with aging (DeVries et al., 2024) and reported that with increasing age, synaptic loss occurred, the expression of the pro-pruning factor C1q increased, whereas CD47 expression decreased, ultimately leading to cognitive impairment. Interestingly, microglia maintained stable C1q expression with age. Therefore, understanding the role of CD47/SIRPα in microglial synaptic pruning is crucial for enhancing our understanding of age-related cognitive decline.

In a mouse model of postoperative cognitive dysfunction, the downregulation of CD47 and SIRPα expression in the hippocampus was associated with increased microglial synaptic pruning, leading to synaptic loss and the exacerbation of cognitive impairment. When SIRPα was overexpressed in the hippocampus by adeno-associated virus (AAV) injection, CD47 expression was correspondingly upregulated. Although this strategy reduces microglial synaptic pruning, it fails to ameliorate postoperative synaptic dysfunction and cognitive deficits. These findings further suggest that the overexpression of SIRPα may trigger compensatory mechanisms for microglial synaptic pruning (Shui et al., 2022).

**Figure 3:**
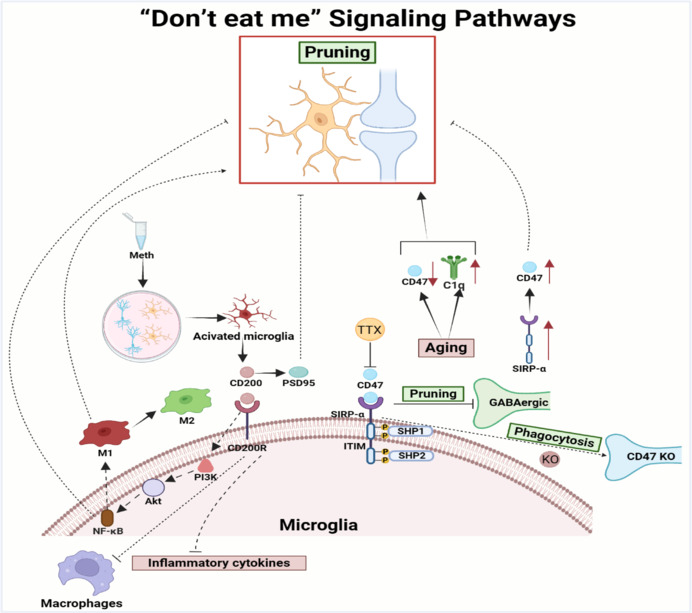
“Don’t eat me” signaling pathways in synaptic pruning. CD47 binding to SIRP-α activates the ITIM motif within SIRP-α, recruiting the tyrosine phosphatases SHP-1/2 to prevent microglial phagocytosis and pruning. TTX-induced suppression of neuronal activity decreases CD47 expression, increasing the susceptibility of synapses to elimination. Aging leads to CD47 downregulation and C1q upregulation, triggering phagocytosis and resulting in cognitive impairment. SIRP-α overexpression increases CD47 expression, further inhibiting synaptic pruning and leading to cognitive deficits. CD200R inhibits monocyte/macrophage activation and inflammatory cytokine release. In a microglia‒hippocampal neuron co-culture model, Meth activated microglia, followed by the upregulation of CD200 and PSD95 expression to prevent synaptic pruning. CD200/CD200R activation initiates PI3K/Akt/NF-κB transcriptional responses, which prevent synaptic pruning and promote a shift in the microglial phenotype from M1 to M2. Created with BioRender.com. Akt: Protein kinase B; C1q: component 1q; CD200: cluster of differentiation 200; CD47: cluster of differentiation 47; GABA: γ-aminobutyric acid; ITIM: immunoreceptor tyrosine-based inhibitory motif; Meth: methamphetamine; NF-κB: nuclear factor kappa-B; PI3K: phosphatidylinositol-3-kinase; PSD95: postsynaptic density protein-95; SIRPα: signal regulatory protein α; TTX: tetrodotoxin.

Collectively, these findings demonstrate that CD47/SIRPα plays a crucial role in preventing excessive microglial pruning, which is highly important for maintaining stable synaptic plasticity. However, the specific molecular mechanisms underlying this process and the impact of CD47/SIRPα on various neurological disorders still require further elucidation. Moreover, considering the potential changes in CD47/SIRPα during aging, an in-depth exploration of its role in age-related neurodegenerative diseases will provide an important theoretical basis for developing novel therapeutic strategies (**Figure 3**).

### CD200/CD200R signaling

CD200 is a transmembrane glycoprotein that exerts immunomodulatory effects across various cell types by inhibiting monocyte/macrophage activation and inflammatory cytokine release through binding to its inhibitory receptor, CD200R (Kassiteridi et al., 2021). In the nervous system, neurons primarily express CD200, whereas microglia predominantly express CD200R (Chamera et al., 2020), suggesting a crucial role for the CD200/CD200R axis in regulating neuroimmune interactions. Notably, CD200R functional deficiency has been demonstrated to inhibit LTP in hippocampal neurons, consequently impairing synaptic plasticity (Manich et al., 2019). To further explore the association between the CD200/CD200R axis and synaptic pruning, Feng et al. (2019) induced CD200 overexpression via hippocampal injection in a mouse model of AD. These authors reported that the upregulation of CD200 not only improved cognition in AD mice but also promoted synaptic plasticity, resulting in increased dendritic spine density. These results suggest that CD200 may function by inhibiting excessive microglial pruning at synapses. Further research has shown that the CD200/CD200R interaction can modulate the balance of pro-inflammatory and anti-inflammatory factors released by microglia, upregulate the expression of synaptic plasticity-related proteins, and increase dendritic spine density, thereby inhibiting post-stroke synaptic loss (Sun et al., 2020). Bravo et al. (2022) co-cultured hippocampal neurons and microglia exposed to methamphetamine and reported upregulated CD200 expression in activated microglia upon neuronal contact. This triggered a neuronal self-protection mechanism, thus preventing abnormal synaptic pruning via increased expression of PSD95. In an aged rat model of anesthesia-induced cognitive dysfunction, reduced CD200/CD200R expression was associated with neuroinflammation and synaptic damage. The administration of the CD200 fusion protein (CD200Fc) ameliorated impaired synaptic function by restoring dendritic spine density and subsequently improved cognitive function. Notably, this study also demonstrated that CD200/CD200R can activate PI3K/Akt/NF-κB, thus promoting the transformation of microglia from the M1 phenotype to the M2 phenotype (Qian et al., 2023). In the context of neuroinflammation, PI3K/Akt/NF-κB activation has been shown to inhibit microglial synaptic pruning (Desale et al., 2021; Xin et al., 2021; Xu et al., 2023a), with M1 microglia recognized as key mediators of synaptic pruning and phagocytosis (Di Liberto et al., 2018). Thus, the state transition of microglia is a critical factor in the CD200/CD200R-mediated prevention of synaptic pruning.

CD200/CD200R is recognized to primarily prevent excessive pruning and elimination of neuronal synapses in microglia. This regulatory role underscores the potential significance of this axis in models of neurological disease and elucidates its mechanism of action on synaptic pruning. Future investigations should focus on elucidating the alterations of this axis in neurodevelopmental disorders and neural circuit dysfunction. Moreover, the CD200/CD200R axis presents a promising therapeutic target for the development of targeted treatment strategies for neurological disorders (**Figure 3**).

The prevailing view holds that the dynamic balance of synaptic pruning is crucial for maintaining healthy brain function. An imbalance in this process not only affects neural network connectivity but also may play a key role in various neurological disorders (Wang et al., 2023). For example, overactivation of the CX3CR1 signaling pathway may lead to increased synaptic elimination, thus disrupting normal synaptic connections and exacerbating neuronal damage (Wu et al., 2023). Therefore, understanding the molecular mechanisms that promote and inhibit synaptic pruning is of paramount importance, although current evidence for many of these molecular mechanisms remains insufficient. In-depth exploration of the complex regulatory networks underlying these mechanisms holds promise for identifying valuable therapeutic strategies and targets for related neurological disorders.

## Research Progress Related to Synaptic Pruning in Common Neurological Disorders

### Alzheimer’s disease

AD is a neurodegenerative disorder characterized by progressive cognitive decline, Aβ formation, and neuronal neurofibrillary tangles. Aβ inhibits synaptic LTP enhancement and enhances LTD, thus exacerbating synaptic dysfunction and disrupting synaptic plasticity (Zhang et al., 2022). In mouse models of AD, Aβ induces C1q localization at synapses in the CA1 region, thus leading to abnormal synaptic pruning and accelerating synaptic loss (Hong et al., 2016). Lau et al. (2020) analyzed brain cell nuclei from AD patients using single-nucleus transcriptomics, revealing several specific cell types associated with microglial activation and synaptic signaling. These authors further observed nonselective synaptic pruning by microglia via activation of the complement system, including C1q. Additionally, these authors reported that the activation of major histocompatibility complex-I promoted CD8^+^ T-cell migration to the brain and IFN-γ release, thus stimulating excessive synaptic pruning by microglia and damaging neural circuits. This is consistent with the findings of Di Liberto et al. (2018). Carpanini et al. (2022) reported in AD mouse models that the activation of C1q and C3b/iC3b to form a membrane attack complex (MAC) exacerbated synaptic pruning. Aloi et al. (2023) reported that knocking out microglial-specific miR-155 in AD mice increased the pruning of excitatory synapses while reducing the pruning of inhibitory synapses, thus leading to an imbalance in synaptic pruning that further aggravated neural network connectivity disorders in AD. These findings indicate that miRNAs also play a role in synaptic pruning in AD by regulating microglia (**Table 1**).

**Table 1:** Mechanisms leading to synaptic loss in various neurological disorders

Disease	Mechanism	Technology	Result	Reference
AD	Aβ induces C1q expression and mediates microglial synaptic pruning; MHC-I activation promotes CD8^+^ T-cellT cell release of IFN-γ, stimulating microglial synaptic pruning; complement factors form the MAC, exacerbating synaptic pruning.	Transgenic animal models; construction of AD mouse models; electrophysiological experiments; single-nucleus transcriptomics.	Excessive microglial pruning leads to synaptic loss, ultimately resulting in neurological dysfunction.	Stevens et al., 2007; Desale et al., 2021; Zhang et al., 2022
	Deletion of microRNA-155 in microglia results in enhanced excitatory synaptic pruning and reduced inhibitory synaptic pruning.	Gene knockout studies; electrophysiological experiments.	Imbalanced synaptic pruning leads to neural network connectivity disorders.	Lau et al., 2020
	The C5a-C5aR1 signaling axis promotes excessive synaptic pruning by microglia.	Transgenic animal models; electrophysiological experiments; golgi staining.	Synaptic loss exacerbates cognitive impairment in AD mice.	Carpanini et al., 2022
ASD	The downregulation of Gprasp2 expression is associated with enhanced mGluR5-mediated LTD.	Gene knockout studies; electrophysiological experiments; behavioral experiments; golgi staining.	Synaptic connections weaken and synapses are lost.	Yao et al., 2022
	The observed decrease in GLS1 expression, reduced complement factor expression, and lowered microglial activity.	Transgenic animal models; electrophysiological experiments; behavioral experiments; golgi staining.	Synaptic pruning is inhibited.	Ji et al., 2023
	Autophagy signaling is dysregulated, characterized by downregulation of LC3-II expression, loss of Atg7 expression, and reduced mTOR activity.	Transgenic animal models; gene knockout studies; behavioral experiments; golgi staining.	Dendritic spine density increases, indicating synaptic pruning dysfunction.	Tang et al., 2014; Kim et al., 2017
	Pten expression is downregulated, C1q is activated, and microglia are activated.	Transgenic animal models.	Synaptic pruning function is enhanced.	Sarn et al., 2021
SCZ	The ErbB4 splice variant changes from JM-b to JM-a, and excitatory synaptic input to PV-positive interneurons is reduced.	Layer-specific RNA extraction and PCR.	Synaptic connections weaken and synaptic pruning is dysfunctional.	Chung et al., 2017
	C4 increases, and microglia are overactivated.	Gene knockout studies; *in utero* electroporation; electrophysiological experiments; behavioral experiments.	Synaptic loss occurs, excitatory synapses decrease, and microglial synaptic phagocytosis ability is enhanced.	Sekar et al., 2016; Germann et al., 2021; Mou et al., 2022
	C4A overexpression enhances microglial phagocytic function.	Transgenic animal models; behavioral experiments; golgi staining.	Synaptic loss occurs due to excessive synaptic pruning by microglia.	Comer et al., 2020
MS	C1q and C3 expression is upregulated, and microglia are activated.	Gene knockout studies; construction of MS mouse models; behavioral experiments.	Synaptic loss occurs as microglia phagocytose synapses.	Reich et al., 2018; Hammond et al., 2020
	Cortical injection of IFN-γ and tumor necrosis factor-α leads to local calcium accumulation and microglial activation.	Construction of MS mouse models; cranial calcium imaginG; golgi staining.	Microglia excessively prune calcium-overloaded synapses, damaging cortical neural circuits.	Jafari et al., 2021
Cerebral ischemia	BDNF released by activated microglia mediates the loss of glutamatergic and GABAergic synapses.	Transgenic animal models; construction of MCAO mouse models; construction of OGD cell models; golgi staining.	Synaptic loss occurs, BDNF promoter IV is dysfunctional, and synaptic pruning is abnormal.	Cramer et al., 2022; Xu et al., 2023b
	MEGF10 and MERTK mediate synaptic phagocytosis by microglia and astrocytes.	Transgenic animal models; construction of MCAO mouse models; *in utero* electroporation; golgi staining; behavioral experiment.	Increased synaptic loss leads to poor disease prognosis.	Shi et al., 2021
	Gephyrin in microglia regulates the release of proBDNF and mBDNF, which further mediates the loss of glutamatergic and GABAergic synapses through p75NTR and TrkB receptors.	Transgenic animal models; construction of MCAO mouse models; construction of OGD cell models; electrophysiological experiments.		Cramer et al., 2022
	ANXA1 overexpression enhances the interaction between CX3CL1 and CX3CR1, and promotes CX3CR1 expression on microglia.	Construction of TIA mouse models; electrophysiological experiment; behavioral experiment; golgi staining.	Synaptic loss occurs as microglia excessively prune synapses.	Zheng et al., 2022

AD: Alzheimer’s disease; ASD: autism spectrum disorder; BDNF: brain-derived neurotrophic factor; C1q: component 1q; GLS1: glutaminase 1; IFN: interferon; LTD: long-term depression; MCAO: middle cerebral artery occlusion; mGluR: metabotropic glutamate receptor; MS: multiple sclerosis; OGD: oxygen and glucose deprivation; SCZ: schizophrenia; TIA: transient ischemic attack.

Although abnormal pruning by microglia exacerbates synaptic dysfunction, research also suggests that intervention with complement factors may also be an effective strategy for treating AD (Hong et al., 2016). In mouse models of AD, the GPCR family member C5aR1 in the hippocampus was found to promote excessive synaptic pruning by microglia. The knockout of C5aR1 resulted in the recovery of long-term and short-term synaptic plasticity after 10 months and prevented excessive pruning. The use of PMX205 to inhibit C5a-C5aR1 activity reduces presynaptic over-pruning and rescues cognitive impairment caused by synaptic loss (Gomez-Arboledas et al., 2024). These findings suggest that C5aR1 may represent a potential therapeutic target for correcting abnormal synaptic pruning in AD. Furthermore, synaptic loss and dysfunction, microglial activation, and increased C1qA expression were observed in the hippocampus and cortex of FAD4T model mice. The intranasal administration of oxytocin reduces C1qA expression and alleviates cognitive impairment (Zhang et al., 2024a). Although this study did not directly demonstrate C1qA-mediated microglial synaptic pruning, whether the therapeutic effect of oxytocin corrects excessive pruning by microglia in the context of AD to achieve neuroprotective function requires further investigation. Furthermore, inhibiting C7 or knocking out C6 to prevent MAC formation has been shown to prevent synaptic loss in mouse models of AD (Carpanini et al., 2022). The treatment of microglia with soluble Aβ oligomers has been shown to promote excessive synaptic phagocytosis and reduce CD47 expression, thus eliminating the instruction to inhibit pruning. Lentivirus-mediated EF1a SIRPα overexpression has been shown to attenuate the phagocytosis of synaptosomes by microglia upon treatment with Aβ oligomers (Ding et al., 2021).

These findings suggest that identifying and intervening with key molecules in the complement system, as well as monitoring abnormal synaptic pruning processes by microglia in AD, could provide insights for developing targeted therapies against abnormal synaptic pruning in AD.

### Autism spectrum disorder

ASD is a neurodevelopmental disorder caused by both genetic and environmental factors and is characterized by social impairments, repetitive behaviors and cognitive dysfunction. Research has shown that the changes in high and low connectivity in the brain networks of ASD patients are closely related to synaptic pruning defects (Bourgeron, 2015; Piochon et al., 2016). The main molecular mechanisms underlying the pathogenesis of ASD are abnormal dendritic spine morphology and increased density, as well as protein dysfunction in these regions (Lo and Lai, 2020).

Glutamate, the main excitatory neurotransmitter in the nervous system, participates in the regulation of cognition, emotion, and synaptic plasticity. Abnormalities in the glutamate signaling pathway are thought to be associated with behavioral abnormalities and cognitive impairments in individuals with ASD (Nisar et al., 2022). Moreover, the glutamate signaling pathway plays an important role in regulating dendritic spine morphology and number (Yao et al., 2022). GPCR-associated sorting protein 2 (Gprasp2) plays a role in regulating metabotropic glutamate receptor activity and is downregulated in the brains of ASD patients. After Gprasp2 was knocked out in mouse brains, researchers reported weakened synaptic connections in the hippocampus, impaired transition of dendritic spines to mature states, enhanced mGluR5-mediated LTD, and ASD-like behaviors. The mGluR5 inhibitor MPEP can prevent the reduction in dendritic spine density induced by reduced Gprasp2 expression (Edfawy et al., 2019). These findings reveal that the interaction between Gprasp2 and glutamate may affect synaptic pruning and the number and state of dendritic spines. Glutaminase 1 (GLS1) is an enzyme that catalyzes the hydrolysis of glutamine to glutamate. Previous studies revealed that GLS1 expression was reduced in the peripheral blood and brain of AD patients. Furthermore, the knockout of GLS1 in mouse brains resulted in ASD-like behaviors and impaired microglial synaptic pruning in the mPFC, thus leading to an imbalance between excitatory and inhibitory synapses. In addition, LPS treatment enhances microglial synaptic pruning ability and improves abnormal behaviors (Ji et al., 2023).

Shank3 is a major postsynaptic scaffold protein that plays a crucial role in regulating synaptic plasticity and glutamate receptor signaling. In Shank3-deficient mouse models, microglial activation and reduced dendritic spine numbers were observed in the hippocampus, and the resulting mice exhibited ASD-like behaviors (Urrutia-Ruiz et al., 2022). The Shank-GKAP-PSD95 complex can anchor the mGluR-Homer1a complex at synapses and activate the mTOR signaling pathway (Bockaert et al., 2021). The mTOR signaling pathway is also essential for regulating synaptic pruning (Zhang et al., 2021b). Tang et al. (2014) demonstrated that the brains of ASD patients presented with synaptic pruning defects, dysregulation of mTOR autophagy signaling, and downregulation of LC3-II expression. These changes suggest that lower levels of mTOR activity may lead to increased dendritic spine density, whereas normal autophagy signaling can mediate the synaptic pruning process. Atg7 is crucial for autophagy function. Kim et al. (2017) created a mouse model of ASD by knocking out Atg7 and reported increased dendritic spine density and defects in synaptic refinement. Further knockout of Atg7 in microglia cultured with hippocampal neurons resulted in increased synaptic numbers and immature dendritic spines, indicating that autophagy dysfunction impairs the ability of microglia to prune synapses. Pagani et al. (2021) used resting-state functional magnetic resonance imaging and functional connectivity analysis to discover stronger brain functional connectivity in ASD mice. This phenomenon may be caused by a synaptic excitation–inhibition imbalance due to excessive dendritic spine numbers, which can be rescued by inhibiting mTOR. Phosphatase and tensin homolog (Pten) dysfunction is closely related to the pathogenesis of ASD. Sarn et al. (2021) constructed a mouse model of ASD by inducing mutations in the Pten gene and reported that a 50% reduction in Pten expression in the cerebral cortex unexpectedly promoted microglial activation and enhanced synaptic pruning function. Further research revealed the upregulation of complement factor C1q expression. These findings suggest that Pten mediates microglial synaptic pruning via complement signaling (**Table 1**); however, these authors did not explore whether this pruning occurred excessively. Notably, Pten is a negative regulator of mTOR, and silencing its expression can activate mTOR (Park et al., 2008). Therefore, mTOR and its autophagy pathway, or other related molecular pathways, are crucial in regulating synaptic pruning in ASD. However, the function of mTOR needs to be investigated further.

In conclusion, synaptic pruning plays a central role in the pathological mechanisms of ASD. Key molecular targets, such as Gprasp2, GLS1, Shank3, and mTOR, play important roles in regulating glutamate signaling in the nervous system, dendritic spine morphology and density, and glial cell synaptic pruning. Future research should focus on the in-depth exploration of the specific mechanisms of action of these molecules in synaptic pruning and how they may facilitate the early diagnosis and prevention of ASD.

### Schizophrenia

SCZ is a complex mental disorder primarily characterized by abnormalities in perception, emotion and behavior. Research has indicated that neural network connectivity disorders and abnormal neural activity in the dlPFC play central roles in the pathophysiology of SCZ (Smucny et al., 2022). Neurodevelopmental disorders are the most prominent features that cause working memory deficits in SCZ patients, with layer 3 pyramidal neurons (L3PNs) in the dlPFC playing a crucial role in regulating working memory (Schoonover et al., 2024). Recent studies have observed synaptic pruning in the L3PNs of adolescent monkeys and reported that the strength and distribution of excitatory synapses remain stable. This suggests that weak or immature synapses are not pruned and that selective pruning of weak synapses may be beneficial for improving specific memory functions but may also affect processes used for performing other tasks (Gonzalez-Burgos et al., 2023). Therefore, understanding this pruning pattern is important if we are to identify the causes of abnormal synaptic pruning in SCZ patients. Chung et al. (2017) compared synaptic pruning levels before and after adolescence and reported that excitatory synapses located on parvalbumin (PV)-positive interneurons underwent significant pruning during adolescence, resulting in increased connectivity; this process was mediated by specific splice variants of erb-b2 receptor tyrosine kinase 4 (ErbB4). In the dlPFC of SCZ patients, the ErbB4 splice variant switches from JM-b to JM-a, directly leading to reduced excitatory synaptic input to PV-positive interneurons and impaired synaptic connectivity (Chung et al., 2016). These findings suggest that the transformation of ErbB4 splice variants in SCZ may be a factor affecting excitatory synaptic pruning (**Table 1**). Furthermore, neuregulin 1 (NRG1), a ligand of ErbB4, is expressed at increased levels in the mPFC of a mouse model of SCZ, whereas the number of PV-positive interneurons is significantly reduced. Following NRG1 knockout, GABA maturation defects in PV-positive interneurons can be improved, thus promoting enhanced synaptic connectivity and alleviating SCZ-like behaviors (Dong et al., 2022). Considering that NRG1 can bind to JM-a (Law et al., 2007), this interaction may affect synaptic pruning and weaken synaptic connections. Therefore, ErbB4-NRG1 may represent a key signal regulating PV interneuron development and synaptic pruning.

Multiple studies have shown that the pathological mechanism underlying SCZ is associated with the expression of complement factors. When overexpressed, these factors can induce excessive microglial activation, leading to the overpruning of synapses (Germann et al., 2021). *In vitro* experiments using cells from SCZ patients revealed that increased C4 expression was proportional to the risk of developing SCZ (Sekar et al., 2016). Comer et al. (2020) used in utero electroporation to overexpress C4 in the mPFC of fetal mice, thus promoting synaptic loss in L2/3 neurons of the mPFC. Patch-clamp techniques further revealed a reduction in excitatory synapses after C4 overexpression, further reducing the connectivity of cortical neurons. In addition, enhanced synaptic phagocytosis by microglia was observed, and mice exhibited SCZ-like behaviors (Comer et al., 2020). Another study showed that C4A exhibited a stronger ability to bind to synapses and could rescue impaired synaptic refinement in C4 KO mice. However, the overexpression of C4A was previously shown to increase the degree of microglial pruning of synapses in the mPFC, resulting in SCZ-like behavioral abnormalities (Yilmaz et al., 2021). Interestingly, in the subventricular zone of SCZ patients, C4 expression has been detected in astrocytes, neurons, and ependymal cells but not in microglia (Mou et al., 2022). These findings suggest that C4 may play different roles in different brain regions or cell types.

Recent research suggests that the tetracycline antibiotic minocycline has therapeutic potential for SCZ. In the adolescent frontal cortex, minocycline acts by inhibiting excessive complement-mediated synaptic pruning by microglia, thereby preventing the onset of SCZ (Jones et al., 2020). The findings of this study indicate that the onset and development of SCZ is a complex process involving genetic factors, immune responses, changes in neuroplasticity and psychological changes. Moreover, although current basic research has made some progress in developing animal models of SCZ, it is still insufficient to fully explain the pathological changes that occur during adolescent brain development. A deeper understanding of the pathogenesis of SCZ, especially how the complement system affects the excessive synaptic pruning of microglia during adolescent brain development, may allow us to extract valuable therapeutic targets from this process and provide a theoretical basis and practical methods for the intervention and treatment of SCZ.

### Multiple sclerosis

Multiple sclerosis (MS) is an autoimmune disease that is characterized primarily by inflammatory demyelination of the white matter in the nervous system, leading to abnormalities in emotion, sensation, cognition and motor function. Research has revealed widespread synaptic loss in the cortex of MS patients (Jürgens et al., 2016). Furthermore, research has shown that astrocytes, microglia and oligodendrocyte precursor cells are located at pathological plaques in MS (Reich et al., 2018). Iron aggregation occurs within microglia in some MS plaques; these plaques are referred to as “paramagnetic foci.” Complement proteins C1q and C3 are expressed at high levels in the microglia of pathological plaques in MS (Absinta et al., 2021). These mechanisms may promote excessive synaptic pruning by microglia, thereby accelerating synaptic dysfunction. Therefore, activation of the complement system and glial cells plays a regulatory role in abnormal synaptic pruning in the context of MS (**Table 1**). Experimental autoimmune encephalomyelitis (EAE) serves as an efficient animal model for MS. Hammond et al. (2020) reported the upregulation of C1q and C3 in the hippocampus of EAE mice. C3 knockout, but not C1q knockout, was also shown to prevent microglial activation and alleviate hippocampal synaptic loss, thus improving learning and memory abilities in EAE mice. Werneburg et al. (2020) induced AAV9-mediated expression of the C3 inhibitor Crry in both eyes of a mouse model of MS and reported that this practice inhibited microglial phagocytosis in synapses of the LGN, thereby restoring visual function. This highlights the importance of C3 in regulating synaptic pruning in the context of MS. Jafari et al. (2021) constructed a mouse model of MS via the stereotaxic injection of IFN-γ and tumor necrosis factor-α into the cortex and reported widespread synaptic loss in the cortex. Further research revealed that these effects were associated with local calcium accumulation in synapses and that activated microglia tended to excessively prune calcium-overloaded synapses, thus exacerbating neural circuit damage in the cortex of MS mice. Therefore, monitoring the calcium content in the cortical synapses of MS patients may be an effective strategy for preventing excessive pruning and maintaining cortical functional network functionality. The main pathological manifestation of MS is demyelination; MERTK-mediated microglial activation, migration and phagocytosis are all necessary for myelin reformation and the recovery of neurological function (Shen et al., 2021). Previous studies have shown that MERTK is an important regulatory molecule for microglial synaptic pruning (Chung et al., 2013; Faust et al., 2021). Therefore, MERTK may be a valuable intervention target for preventing the progression of MS.

Given that CD47 is an important molecule in preventing excessive synaptic pruning (Lehrman et al., 2018; Ding et al., 2021; DeVries et al., 2024), we could hypothesize that CD47 could inhibit excessive synaptic pruning in MS. Han et al. demonstrated that in patients with MS, there was a downregulation of CD47 expression in the brain, which was associated with increased phagocytic activity and demyelination. However, in a mouse model of EAE, while CD47 knockout inhibited peripheral immune cell activation, paradoxically, blocking CD47 during the disease exacerbation phase exacerbated the inflammatory response. Conversely, blocking CD47 during the stable phase ameliorated disease progression, suggesting that CD47 may serve as a bidirectional regulatory target potentially by modulating immune responses. Additionally, timely intervention at different stages of the disease may have positive implications (Han et al., 2012). Zhao et al. (2023) developed a CD47 peptide-modified nFTY nanoparticle (clnFTY nanoparticle) that exhibited superior drug delivery properties and effectively inhibited the progression of EAE and the release of inflammatory factors. We hypothesize that nanoparticles possessing CD47 biological functionality might achieve neuroprotection by inhibiting microglial activation, thereby preventing their participation in synaptic pruning. However, it is important to note that the effective phagocytic function of microglia is beneficial for myelin regeneration in MS. Gitik et al. investigated mouse models of peripheral nerve and spinal cord injury following CD47 knockout and reported that Schwann cells can clear myelin debris more rapidly, thus accelerating postinjury repair. Although the pathological mechanisms of spinal cord injury and MS differ, both involve demyelination phenomena. These findings suggest that when CD47 is used as a therapeutic approach, its impact on myelin regeneration in MS should be evaluated on the basis of the specific characteristics of different diseases.

Research on MS has focused predominantly on immune regulatory mechanisms, inflammatory responses, and complement pathways. However, our understanding of the specific molecular mechanisms regulating synaptic pruning during the disease process remains limited. Future research should focus on an in-depth analysis of the genes and molecular pathways that lead to abnormal synaptic pruning in MS.

### Cerebral ischemia

With the increasing aging population, stroke has become one of the diseases with one of the highest global incidences. Ischemic stroke, the most common type of stroke, accounts for approximately two-thirds of all cases and is predominantly caused by atherosclerosis, vascular stenosis, and thrombosis. These processes can lead to interruption of the local blood supply to the brain and subsequent hypoxia. In this state, microglial activation triggered by excitotoxicity, oxidative stress and mitochondrial dysfunction releases inflammatory factors, thus exacerbating brain damage. Cerebral ischemia can lead to a significant reduction in the number of dendritic spines (Wang et al., 2016). Recent research revealed that poststroke limb function decline in mice is closely related to reduced sensory responses in the damaged area, a phenomenon caused by the inhibition of GABAergic synaptic function (Kokinovic and Medini, 2018). The signaling pathway mediated by BDNF and TrkB is a key mechanism regulating synapse formation. Studies have shown that BDNF containing exon IV can promote dendritic spine density and synaptic numbers, whereas BDNF dysfunction leads to reduced synaptic numbers (Wang et al., 2022a; Bach et al., 2024). Using a mouse model of middle cerebral artery occlusion (MCAO), researchers reported that BDNF was released by microglia and mediated the loss of glutamatergic and GABAergic synapses (Cramer et al., 2022). Therefore, we can hypothesize that under cerebral ischemia, the BDNF promoter IV may be damaged, not only affecting the normal process of synaptic pruning but also potentially causing a reduction in GABAergic synapses (Xu et al., 2023b), further exacerbating the neuronal damage caused by cerebral ischemia. Multiple EGF-like domain 10 (MEGF10) is a major regulatory protein that mediates the astrocytic phagocytosis of synapses and is crucial for maintaining synaptic plasticity and normal connectivity (Lee et al., 2021). Shi et al. (2021) reported that in reactive microglia caused by ischemic stroke, the activation of MEGF10 and MERTK can mediate the excessive phagocytosis of synapses by microglia and exacerbate neuronal damage. Therefore, MEGF10 and MERTK can be viewed as potential targets for synaptic phagocytosis; inhibiting their expression may be beneficial for the recovery of neurological function after cerebral ischemia (**Table 1**). However, we need to ascertain whether this affects the phagocytosis of abnormal synapses, thus impacting the degree of synaptic refinement. In conclusion, the activation state of microglia is a target for regulating the degree of brain damage and synaptic function.

Previous *in vitro* OGD experiments simulating cerebral ischemia have shown that proBDNF and p75NTR can lead to synaptic reduction (Woo et al., 2005; Wang et al., 2022b). proBDNF and BDNF can induce synaptic loss via p75NTR and TrkB signaling, respectively. Previous researchers added TrkB-Fc to hippocampal neurons and observed the downregulation of phosphorylated TrkB and BDNF expression, thus rescuing OGD-induced synaptic loss (Cramer et al., 2022). Furthermore, hydrogen-rich saline has been shown to prevent synaptic loss by promoting microglial transformation to the M2 phenotype and by inhibiting the activation of complement signal-related pathways (Chu et al., 2019). In summary, these data suggest that in cerebral ischemia, synaptic loss is caused primarily by microglial activation-mediated synaptic phagocytosis by signaling pathways such as MEGF10 and BDNF. These processes are abnormal and may lead to disruption of brain neural circuits and reduced synaptic refinement. Therefore, interventions targeting the signaling pathways involved in microglial activation may help in the development of therapeutic strategies for synaptic loss in the context of ischemic stroke.

## Recent Advances in Emerging Imaging Techniques Applied to Synaptic Pruning Research

Synaptic dysfunction represents a critical factor in neural circuit disruption and brain malfunction, making understanding the mechanisms of abnormal synaptic function essential. Assessment of synaptic morphology, intersynaptic connectivity patterns, and temporal dynamics provides crucial insights into neuronal communication efficacy. Despite challenges in directly observing the complex distribution of neurons and microstructures in brain tissue, recent research (Michalska et al., 2024) has developed the “Comprehensive Analysis of Tissues across Scales” technique. This approach employs stimulated emission depletion (STED) super-resolution optical microscopy to map synaptic inputs, track complex axons and synaptic connections in three-dimensional tissue, reconstruct individual synapses with nanoscale resolution, and identify synaptic clefts to generate synaptic connectivity maps. This technique enables tissue analysis across spatial dimensions from millimeters to nanometers, facilitating longitudinal tracking of developmental synaptic pruning, comparative analysis of synaptic density and morphological alterations between physiological and pathological states, investigation of activity-dependent synaptic refinement, and evaluation of pharmacological or genetic interventions on pruning mechanisms. Additionally, Son et al. (2024) developed a technique called “synapshot”, which uses dimerization-dependent fluorescent proteins (ddFPs) conjugated with synaptic adhesion molecules, specifically neurexin and neuroligin, to monitor reversible and bidirectional structural modifications at synaptic junctions in real time. Despite potential signal attenuation following formaldehyde fixation, this methodology effectively discriminates synaptic functional activity through differential green and red fluorescent protein expression, making it particularly suitable for real-time visualization of synaptic dynamics in living cells and intact brain preparations. Notably, dendritic spine atrophy was observed subsequent to diminished “synapshot” signal intensity. Under physiological conditions, microglia can prune abnormal synapses to promote normal neural circuits. In pathological contexts, several critical questions warrant investigation: (1) Can these imaging techniques reliably detect and characterize abnormal dendritic spine morphology? (2) Can dual fluorescent protein labeling enable differential identification of normal versus pathological synaptic populations and their respective connectivity patterns? (3) Can quantitative analyses of normal versus abnormal synaptic populations, integrated with assessments of microglial activation and associated molecular mechanisms, elucidate synaptic pruning processes? Complementary macroscale approaches include positron emission tomography (PET) with radiolabeled tracers such as ^18^F-fluorodeoxyglucose (^18^F-FDG) to assess regional brain metabolism mediated through glutamatergic synapses and glucose utilization. In AD, diminished ^18^F-FDG uptake is correlated with reduced synaptic density and impaired functional connectivity between brain regions (Fessel, 2021; Zhang et al., 2023b). Similarly, resting-state functional magnetic resonance imaging (rs-fMRI) has revealed altered connectivity patterns in individuals with ASD, specifically hyperconnectivity in pediatric patients and murine models, which is indicative of synaptic hyperactivity and aberrant synaptic connectivity (Pagani et al., 2021).

The integration of these multimodal detection techniques enables comprehensive characterization of synaptic pruning mechanisms—from microscopic alterations in individual synapses to macroscopic functional connectivity networks—providing novel perspectives and methodological approaches for identifying and therapeutically targeting abnormal synaptic pruning in neuropsychiatric and neurodegenerative disorders, including SCZ and AD. This approach enhances our understanding of synaptic pruning mechanisms across temporal and spatial dimensions.

## Limitations

This review explores the spontaneous and experience-dependent neuronal activities that trigger synaptic pruning, yet the specific molecular mechanisms influencing the “eat-me” and “don’t eat-me” signaling pathways remain unclear. Current synaptic pruning research relies primarily on morphological indicators such as dendritic spine density quantification or synaptic protein expression levels. Although imaging technologies can observe synaptic morphological changes at the nanoscale, methodological approaches for directly observing and quantitatively analyzing the dynamic process of synaptic pruning are lacking. This technical limitation constrains in-depth investigations of the dynamic regulatory mechanisms of synaptic pruning and their interaction networks with synaptic plasticity. Glial cells play crucial roles in the synaptic pruning process. They not only participate in clearing redundant synapses and modulating neuronal activity through the secretion of various neurotrophic factors and cytokines but, in certain circumstances, may also eliminate functionally normal synapses. Consequently, a key direction for future research is to clarify how glial cells precisely identify and prune specific synapses and to characterize the similarities and differences in their signaling interactions with neurons across different brain regions and developmental periods. This review has not yet thoroughly discussed the specific functions of astrocytes and oligodendrocytes in synaptic pruning. Finally, both hyperactivation and insufficient function of synaptic pruning are closely associated with the pathophysiological mechanisms of various neurological disorders, yet effective intervention strategies are currently lacking. In future research, we should focus on identifying more molecular targets and developing multi-target combination therapeutic strategies to correct abnormal synaptic pruning processes in neurological diseases.

## Conclusions and Perspectives

In this review, we elucidate how spontaneous neural activity and experience-dependent factors trigger synaptic pruning, emphasizing the critical role of these processes in synaptic refinement and neural circuit optimization. We conducted a comprehensive analysis of the molecular signaling cascades governing “eat-me” and “don’t-eat-me” signals that regulate synaptic pruning, elucidating their downstream effectors and regulatory mechanisms. Additionally, we critically evaluate the role of synaptic pruning in AD, ASD, SCZ, MS, and cerebral ischemia while comprehensively reviewing the current state of research on related molecular mechanisms. Moreover, considering the limitations of conventional optical imaging techniques in detecting subtle synaptic changes, we explore recent advancements in optical and imaging technologies and their applications in synaptic pruning research. Nevertheless, a critical challenge persists in accurately differentiating between pathological synaptic loss and normal developmental synaptic pruning in the context of neurological disorders. The intricate nature of synaptic structures and the complexity of their functions present numerous challenges for current research in this field. Future advancements in high-resolution imaging techniques and the development of specific synaptic molecular markers will facilitate the distinction between pathological synaptic loss and normal synaptic pruning, potentially enhancing diagnostic accuracy and therapeutic interventions for synaptic abnormalities in neurological disorders.

## Data Availability

*Not applicable*.

## References

[R1] Aarse J, Herlitze S, Manahan-Vaughan D (2016). The requirement of BDNF for hippocampal synaptic plasticity is experience-dependent. Hippocampus.

[R2] Abokyi S, Tse DY (2025). Age-related driving mechanisms of retinal diseases and neuroprotection by transcription factor EB-targeted therapy. Neural Regen Res.

[R3] Absinta M, Maric D, Gharagozloo M, Garton T, Smith MD, Jin J, Fitzgerald KC, Song A, Liu P, Lin JP, Wu T, Johnson KR, McGavern DB, Schafer DP, Calabresi PA, Reich DS (2021). A lymphocyte-microglia-astrocyte axis in chronic active multiple sclerosis. Nature.

[R4] Almasieh M, Faris H, Levin LA (2022). Pivotal roles for membrane phospholipids in axonal degeneration. Int J Biochem Cell Biol.

[R5] Aloi MS, Prater KE, Sánchez REA, Beck A, Pathan JL, Davidson S, Wilson A, Keene CD, de la Iglesia H, Jayadev S, Garden GA (2023). Microglia specific deletion of miR-155 in Alzheimer’s disease mouse models reduces amyloid-β pathology but causes hyperexcitability and seizures. J Neuroinflammation.

[R6] Angelopoulou E, Paudel YN, Shaikh MF, Piperi C (2020). Fractalkine (CX3CL1) signaling and neuroinflammation in Parkinson’s disease: Potential clinical and therapeutic implications. Pharmacol Res.

[R7] Antunes FM, Rubio ME, Kandler K (2020). Role of GluA3 AMPA receptor subunits in the presynaptic and postsynaptic maturation of synaptic transmission and plasticity of endbulb-bushy cell synapses in the cochlear nucleus. J Neurosci.

[R8] Araque A, Parpura V, Sanzgiri RP, Haydon PG (1999). Tripartite synapses: glia, the unacknowledged partner. Trends Neurosci.

[R9] Assali A, Gaspar P, Rebsam A (2014). Activity dependent mechanisms of visual map formation--from retinal waves to molecular regulators. Semin Cell Dev Biol.

[R10] Avarlaid A, Falkenberg K, Lehe K, Mudò G, Belluardo N, Di Liberto V, Frinchi M, Tuvikene J, Timmusk T (2024). An upstream enhancer and MEF2 transcription factors fine-tune the regulation of the Bdnf gene in cortical and hippocampal neurons. J Biol Chem.

[R11] Bach SV, Bauman AJ, Hosein D, Tuscher JJ, Ianov L, Greathouse KM, Henderson BW, Herskowitz JH, Martinowich K, Day JJ (2024). Distinct roles of Bdnf I and Bdnf IV transcript variant expression in hippocampal neurons. Hippocampus.

[R12] Baldinotti R, Pauzin FP, Fevang H, Ishizuka Y, Bramham CR (2025). A nanobody-based proximity ligation assay detects constitutive and stimulus-regulated native Arc/Arg3.1 oligomers in hippocampal neuronal dendrites. Mol Neurobiol.

[R13] Baranov SV, Jauhari A, Carlisle DL, Friedlander RM (2021). Two hit mitochondrial-driven model of synapse loss in neurodegeneration. Neurobiol Dis.

[R14] Barnes SJ, Franzoni E, Jacobsen RI, Erdelyi F, Szabo G, Clopath C, Keller GB, Keck T (2017). Deprivation-induced homeostatic spine scaling in vivo is localized to dendritic branches that have undergone recent spine loss. Neuron.

[R15] Bockaert J, Perroy J, Ango F (2021). The complex formed by group i metabotropic glutamate receptor (mGluR) and Homer1a plays a central role in metaplasticity and homeostatic synaptic scaling. J Neurosci.

[R16] Bonzano S, Dallorto E, Molineris I, Michelon F, Crisci I, Gambarotta G, Neri F, Oliviero S, Beckervordersandforth R, Lie DC, Peretto P, Bovetti S, Studer M, De Marchis S (2023). NR2F1 shapes mitochondria in the mouse brain, providing new insights into Bosch-Boonstra-Schaaf optic atrophy syndrome. Dis Model Mech.

[R17] Bourgeron T (2015). From the genetic architecture to synaptic plasticity in autism spectrum disorder. Nat Rev Neurosci.

[R18] Bravo J, Ribeiro I, Terceiro AF, Andrade EB, Portugal CC, Lopes IM, Azevedo MM, Sousa M, Lopes CDF, Lobo AC, Canedo T, Relvas JB, Summavielle T (2022). Neuron-microglia contact-dependent mechanisms attenuate methamphetamine-induced microglia reactivity and enhance neuronal plasticity. Cells.

[R19] Brelstaff J, Tolkovsky AM, Ghetti B, Goedert M, Spillantini MG (2018). Living neurons with Tau filaments aberrantly expose phosphatidylserine and are phagocytosed by microglia. Cell Rep.

[R20] Burbridge TJ, Xu HP, Ackman JB, Ge X, Zhang Y, Ye MJ, Zhou ZJ, Xu J, Contractor A, Crair MC (2014). Visual circuit development requires patterned activity mediated by retinal acetylcholine receptors. Neuron.

[R21] Camacho-Hernández NP, Peña-Ortega F (2023). Fractalkine/CX3CR1-dependent modulation of synaptic and network plasticity in health and disease. Neural Plast.

[R22] Campelo T, Augusto E, Chenouard N, de Miranda A, Kouskoff V, Camus C, Choquet D, Gambino F (2020). AMPAR-dependent synaptic plasticity initiates cortical remapping and adaptive behaviors during sensory experience. Cell Rep.

[R23] Campos RMP, Barbosa-Silva MC, Ribeiro-Resende VT (2023). A period of transient synaptic density unbalancing in the motor cortex after peripheral nerve injury and the involvement of microglial cells. Mol Cell Neurosci.

[R24] Carlock C, Wu J, Shim J, Moreno-Gonzalez I, Pitcher MR, Hicks J, Suzuki A, Iwata J, Quevado J, Lou Y (2017). Interleukin33 deficiency causes tau abnormality and neurodegeneration with Alzheimer-like symptoms in aged mice. Transl Psychiatry.

[R25] Carpanini SM, Torvell M, Bevan RJ, Byrne RAJ, Daskoulidou N, Saito T, Saido TC, Taylor PR, Hughes TR, Zelek WM, Morgan BP (2022). Terminal complement pathway activation drives synaptic loss in Alzheimer’s disease models. Acta Neuropathol Commun.

[R26] Chamera K, Kotarska K, Szuster-Głuszczak M, Trojan E, Skórkowska A, Pomierny B, Krzyżanowska W, Bryniarska N, Basta-Kaim A (2020). The prenatal challenge with lipopolysaccharide and polyinosinic:polycytidylic acid disrupts CX3CL1-CX3CR1 and CD200-CD200R signalling in the brains of male rat offspring: a link to schizophrenia-like behaviours. J Neuroinflammation.

[R27] Chanaday NL, Kavalali ET (2022). Role of the endoplasmic reticulum in synaptic transmission. Curr Opin Neurobiol.

[R28] Chang CW, Wilkerson JR, Hale CF, Gibson JR, Huber KM (2017). Distinct stages of synapse elimination are induced by burst firing of CA1 neurons and differentially require MEF2A/D. Elife.

[R29] Chidambaram SB, Rathipriya AG, Bolla SR, Bhat A, Ray B, Mahalakshmi AM, Manivasagam T, Thenmozhi AJ, Essa MM, Guillemin GJ, Chandra R, Sakharkar MK (2019). Dendritic spines: Revisiting the physiological role. Prog Neuropsychopharmacol Biol Psychiatry.

[R30] Chu X, Cao L, Yu Z, Xin D, Li T, Ma W, Zhou X, Chen W, Liu D, Wang Z (2019). Hydrogen-rich saline promotes microglia M2 polarization and complement-mediated synapse loss to restore behavioral deficits following hypoxia-ischemic in neonatal mice via AMPK activation. J Neuroinflammation.

[R31] Chung DW, Volk DW, Arion D, Zhang Y, Sampson AR, Lewis DA (2016). Dysregulated ErbB4 splicing in schizophrenia: selective effects on parvalbumin expression. Am J Psychiatry.

[R32] Chung DW, Wills ZP, Fish KN, Lewis DA (2017). Developmental pruning of excitatory synaptic inputs to parvalbumin interneurons in monkey prefrontal cortex. Proc Natl Acad Sci U S A.

[R33] Chung HY (2023). Microglia mediate neurocognitive deficits by eliminating C1q-tagged synapses in sepsis-associated encephalopathy. Sci Adv.

[R34] Chung WS, Clarke LE, Wang GX, Stafford BK, Sher A, Chakraborty C, Joung J, Foo LC, Thompson A, Chen C, Smith SJ, Barres BA (2013). Astrocytes mediate synapse elimination through MEGF10 and MERTK pathways. Nature.

[R35] Combe CL, Gasparini S (2021). I(h) from synapses to networks: HCN channel functions and modulation in neurons. Prog Biophys Mol Biol.

[R36] Comer AL, Jinadasa T, Sriram B, Phadke RA, Kretsge LN, Nguyen TPH, Antognetti G, Gilbert JP, Lee J, Newmark ER, Hausmann FS, Rosenthal S, Liu Kot K, Liu Y, Yen WW, Dejanovic B, Cruz-Martín A (2020). Increased expression of schizophrenia-associated gene C4 leads to hypoconnectivity of prefrontal cortex and reduced social interaction. PLoS Biol.

[R37] Cotman CW, Matthews DA, Taylor D, Lynch G (1973). Synaptic rearrangement in the dentate gyrus: histochemical evidence of adjustments after lesions in immature and adult rats. Proc Natl Acad Sci U S A.

[R38] Cramer T, Gill R, Thirouin ZS, Vaas M, Sampath S, Martineau F, Noya SB, Panzanelli P, Sudharshan TJJ, Colameo D, Chang PK, Wu PY, Shi R, Barker PA, Brown SA, Paolicelli RC, Klohs J, McKinney RA, Tyagarajan SK (2022). Cross-talk between GABAergic postsynapse and microglia regulate synapse loss after brain ischemia. Sci Adv.

[R39] de Arce KP, Ribic A, Chowdhury D, Watters K, Thompson GJ, Sanganahalli BG, Lippard ETC, Rohlmann A, Strittmatter SM, Missler M, Hyder F, Biederer T (2023). Concerted roles of LRRTM1 and SynCAM 1 in organizing prefrontal cortex synapses and cognitive functions. Nat Commun.

[R40] De Luca C, Colangelo AM, Virtuoso A, Alberghina L, Papa M (2020). Neurons, glia, extracellular matrix and neurovascular unit: a systems biology approach to the complexity of synaptic plasticity in health and disease. Int J Mol Sci.

[R41] Del Pino I, Tocco C, Magrinelli E, Marcantoni A, Ferraguto C, Tomagra G, Bertacchi M, Alfano C, Leinekugel X, Frick A, Studer M (2020). COUP-TFI/Nr2f1 orchestrates intrinsic neuronal activity during development of the somatosensory cortex. Cereb Cortex.

[R42] Delmas C, Dalmas E (2018). IL-33 deals with the gray matter. Immunity.

[R43] Desale SE, Chidambaram H, Chinnathambi S (2021). G-protein coupled receptor, PI3K and Rho signaling pathways regulate the cascades of Tau and amyloid-β in Alzheimer’s disease. Mol Biomed.

[R44] DeVries SA, Conner B, Dimovasili C, Moore TL, Medalla M, Mortazavi F, Rosene DL (2024). Immune proteins C1q and CD47 may contribute to aberrant microglia-mediated synapse loss in the aging monkey brain that is associated with cognitive impairment. Geroscience.

[R45] Di Liberto G (2018). Neurons under T cell attack coordinate phagocyte-mediated synaptic stripping. Cell.

[R46] Ding X, Wang J, Huang M, Chen Z, Liu J, Zhang Q, Zhang C, Xiang Y, Zen K, Li L (2021). Loss of microglial SIRPα promotes synaptic pruning in preclinical models of neurodegeneration. Nat Commun.

[R47] Dityatev A, Frischknecht R, Seidenbecher CI (2006). Extracellular matrix and synaptic functions. Results Probl Cell Differ.

[R48] Doma KM, Lewis ED, Barracato JM, Brink LR, Gratson AA, Pandey N, Crowley DC, Evans M (2023). A randomized, double-blind, placebo-controlled, parallel study investigating the efficacy of a whole coffee cherry extract and phosphatidylserine formulation on cognitive performance of healthy adults with self-perceived memory problems. Neurol Ther.

[R49] Dong J, Chen W, Liu N, Chang S, Zhu W, Kang J (2022). NRG1 knockdown rescues PV interneuron GABAergic maturation deficits and schizophrenia behaviors in fetal growth restriction mice. Cell Death Discov.

[R50] Durand GM, Kovalchuk Y, Konnerth A (1996). Long-term potentiation and functional synapse induction in developing hippocampus. Nature.

[R51] Edfawy M, Guedes JR, Pereira MI, Laranjo M, Carvalho MJ, Gao X, Ferreira PA, Caldeira G, Franco LO, Wang D, Cardoso AL, Feng G, Carvalho AL, Peça J (2019). Abnormal mGluR-mediated synaptic plasticity and autism-like behaviours in Gprasp2 mutant mice. Nat Commun.

[R52] Faust TE, Gunner G, Schafer DP (2021). Mechanisms governing activity-dependent synaptic pruning in the developing mammalian CNS. Nat Rev Neurosci.

[R53] Feng D, Huang A, Yan W, Chen D (2019). CD200 dysfunction in neuron contributes to synaptic deficits and cognitive impairment. Biochem Biophys Res Commun.

[R54] Fessel J (2021). Does synaptic hypometabolism or synaptic dysfunction, originate cognitive loss? Analysis of the evidence. Alzheimers Dement (N Y).

[R55] Freria CM, Hall JC, Wei P, Guan Z, McTigue DM, Popovich PG (2017). Deletion of the fractalkine receptor, CX3CR1, improves endogenous repair, axon sprouting, and synaptogenesis after spinal cord injury in mice. J Neurosci.

[R56] Fujimoto S, Leiwe MN, Aihara S, Sakaguchi R, Muroyama Y, Kobayakawa R, Kobayakawa K, Saito T, Imai T (2023). Activity-dependent local protection and lateral inhibition control synaptic competition in developing mitral cells in mice. Dev Cell.

[R57] García-Hernández S, Abe M, Sakimura K, Rubio ME (2017). Impaired auditory processing and altered structure of the endbulb of Held synapse in mice lacking the GluA3 subunit of AMPA receptors. Hear Res.

[R58] Germann M, Brederoo SG, Sommer IEC (2021). Abnormal synaptic pruning during adolescence underlying the development of psychotic disorders. Curr Opin Psychiatry.

[R59] Gheibihayat SM, Cabezas R, Nikiforov NG, Jamialahmadi T, Johnston TP, Sahebkar A (2021). CD47 in the brain and neurodegeneration: an update on the role in neuroinflammatory pathways. Molecules.

[R60] Gitik M, Elberg G, Reichert F, Tal M, Rotshenker S (2023). Deletion of CD47 from Schwann cells and macrophages hastens myelin disruption/dismantling and scavenging in Schwann cells and augments myelin debris phagocytosis in macrophages. J Neuroinflammation.

[R61] Gomez-Arboledas A, Acharya MM, Tenner AJ (2021). The role of complement in synaptic pruning and neurodegeneration. Immunotargets Ther.

[R62] Gomez-Arboledas A, Fonseca MI, Kramar E, Chu SH, Schartz ND, Selvan P, Wood MA, Tenner AJ (2024). C5aR1 signaling promotes region- and age-dependent synaptic pruning in models of Alzheimer’s disease. Alzheimers Dement.

[R63] Gonzalez-Burgos G, Miyamae T, Nishihata Y, Krimer OL, Lewis DA (2023). Strength of excitatory inputs to Layer 3 pyramidal neurons during synaptic pruning in the monkey prefrontal cortex: relevance for the pathogenesis of schizophrenia. Biol Psychiatry.

[R64] Gottschalk WA, Jiang H, Tartaglia N, Feng L, Figurov A, Lu B (1999). Signaling mechanisms mediating BDNF modulation of synaptic plasticity in the hippocampus. Learn Mem.

[R65] Gunner G, Cheadle L, Johnson KM, Ayata P, Badimon A, Mondo E, Nagy MA, Liu L, Bemiller SM, Kim KW, Lira SA, Lamb BT, Tapper AR, Ransohoff RM, Greenberg ME, Schaefer A, Schafer DP (2019). Sensory lesioning induces microglial synapse elimination via ADAM10 and fractalkine signaling. Nat Neurosci.

[R66] Hajipour S, Khombi Shooshtari M, Farbood Y, Ali Mard S, Sarkaki A, Moradi Chameh H, Sistani Karampour N, Ghafouri S (2023). Fingolimod administration following hypoxia induced neonatal seizure can restore impaired long-term potentiation and memory performance in adult rats. Neuroscience.

[R67] Hammond JW, Bellizzi MJ, Ware C, Qiu WQ, Saminathan P, Li H, Luo S, Ma SA, Li Y, Gelbard HA (2020). Complement-dependent synapse loss and microgliosis in a mouse model of multiple sclerosis. Brain Behav Immun.

[R68] Han KS, Cooke SF, Xu W (2017). Experience-dependent equilibration of AMPAR-mediated synaptic transmission during the critical period. Cell Rep.

[R69] Han MH, Lundgren DH, Jaiswal S, Chao M, Graham KL, Garris CS, Axtell RC, Ho PP, Lock CB, Woodard JI, Brownell SE, Zoudilova M, Hunt JF, Baranzini SE, Butcher EC, Raine CS, Sobel RA, Han DK, Weissman I, Steinman L (2012). Janus-like opposing roles of CD47 in autoimmune brain inflammation in humans and mice. J Exp Med.

[R70] Han RT, Vainchtein ID, Schlachetzki JCM, Cho FS, Dorman LC, Ahn E, Kim DK, Barron JJ, Nakao-Inoue H, Molofsky AB, Glass CK, Paz JT, Molofsky AV (2023). Microglial pattern recognition via IL-33 promotes synaptic refinement in developing corticothalamic circuits in mice. J Exp Med.

[R71] Hauswirth AG, Ford KJ, Wang T, Fetter RD, Tong A, Davis GW (2018). A postsynaptic PI3K-cII dependent signaling controller for presynaptic homeostatic plasticity. Elife.

[R72] He D, Xu H, Zhang H, Tang R, Lan Y, Xing R, Li S, Christian E, Hou Y, Lorello P, Caldarone B, Ding J, Nguyen L, Dionne D, Thakore P, Schnell A, Huh JR, Rozenblatt-Rosen O, Regev A, Kuchroo VK (2022). Disruption of the IL-33-ST2-AKT signaling axis impairs neurodevelopment by inhibiting microglial metabolic adaptation and phagocytic function. Immunity.

[R73] He X, Li J, Zhou G, Yang J, McKenzie S, Li Y, Li W, Yu J, Wang Y, Qu J, Wu Z, Hu H, Duan S, Ma H (2021). Gating of hippocampal rhythms and memory by synaptic plasticity in inhibitory interneurons. Neuron.

[R74] Hill TC, Zito K (2013). LTP-induced long-term stabilization of individual nascent dendritic spines. J Neurosci.

[R75] Hong S, Beja-Glasser VF, Nfonoyim BM, Frouin A, Li S, Ramakrishnan S, Merry KM, Shi Q, Rosenthal A, Barres BA, Lemere CA, Selkoe DJ, Stevens B (2016). Complement and microglia mediate early synapse loss in Alzheimer mouse models. Science.

[R76] Hooks BM, Chen C (2006). Distinct roles for spontaneous and visual activity in remodeling of the retinogeniculate synapse. Neuron.

[R77] Hristovska I, Robert M, Combet K, Honnorat J, Comte JC, Pascual O (2022). Sleep decreases neuronal activity control of microglial dynamics in mice. Nat Commun.

[R78] Huttenlocher PR (1990). Morphometric study of human cerebral cortex development. Neuropsychologia.

[R79] Jafari M (2021). Phagocyte-mediated synapse removal in cortical neuroinflammation is promoted by local calcium accumulation. Nat Neurosci.

[R80] Ji C, Tang Y, Zhang Y, Huang X, Li C, Yang Y, Wu Q, Xia X, Cai Q, Qi XR, Zheng JC (2023). Glutaminase 1 deficiency confined in forebrain neurons causes autism spectrum disorder-like behaviors. Cell Rep.

[R81] Jiang T, Li Y, He S, Huang N, Du M, Zhai Q, Pu K, Wu M, Yan C, Ma Z, Wang Q (2023). Reprogramming astrocytic NDRG2/NF-κB/C3 signaling restores the diabetes-associated cognitive dysfunction. EBioMedicine.

[R82] Jones MC, Koh JM, Cheong KH (2020). Synaptic pruning in schizophrenia: does minocycline modulate psychosocial brain development?. Bioessays.

[R83] Jürgens T, Jafari M, Kreutzfeldt M, Bahn E, Brück W, Kerschensteiner M, Merkler D (2016). Reconstruction of single cortical projection neurons reveals primary spine loss in multiple sclerosis. Brain.

[R84] Kaiser N, Pätz C, Brachtendorf S, Eilers J, Bechmann I (2020). Undisturbed climbing fiber pruning in the cerebellar cortex of CX(3) CR1-deficient mice. Glia.

[R85] Kanno T, Nishizaki T, Proia RL, Kajimoto T, Jahangeer S, Okada T, Nakamura S (2010). Regulation of synaptic strength by sphingosine 1-phosphate in the hippocampus. Neuroscience.

[R86] Kassiteridi C, Cole JE, Griseri T, Falck-Hansen M, Goddard ME, Seneviratne AN, Green PA, Park I, Shami AG, Pattarabanjird T, Upadhye A, Taylor AM, Handa A, Channon KM, Lutgens E, McNamara CA, Williams RO, Monaco C (2021). CD200 limits monopoiesis and monocyte recruitment in atherosclerosis. Circ Res.

[R87] Kerschensteiner D (2014). Spontaneous network activity and synaptic development. Neuroscientist.

[R88] Kim HJ, Cho MH, Shim WH, Kim JK, Jeon EY, Kim DH, Yoon SY (2017). Deficient autophagy in microglia impairs synaptic pruning and causes social behavioral defects. Mol Psychiatry.

[R89] Kim SW, Cho KJ (2014). Activity-dependent alterations in the sensitivity to BDNF-TrkB signaling may promote excessive dendritic arborization and spinogenesis in fragile X syndrome in order to compensate for compromised postsynaptic activity. Med Hypotheses.

[R90] Kirkby LA, Sack GS, Firl A, Feller MB (2013). A role for correlated spontaneous activity in the assembly of neural circuits. Neuron.

[R91] Kokinovic B, Medini P (2018). Loss of GABA(B) -mediated interhemispheric synaptic inhibition in stroke periphery. J Physiol.

[R92] Kurematsu C (2022). Synaptic pruning of murine adult-born neurons by microglia depends on phosphatidylserine. J Exp Med.

[R93] Lau SF, Cao H, Fu AKY, Ip NY (2020). Single-nucleus transcriptome analysis reveals dysregulation of angiogenic endothelial cells and neuroprotective glia in Alzheimer’s disease. Proc Natl Acad Sci U S A.

[R94] Law AJ, Kleinman JE, Weinberger DR, Weickert CS (2007). Disease-associated intronic variants in the ErbB4 gene are related to altered ErbB4 splice-variant expression in the brain in schizophrenia. Hum Mol Genet.

[R95] Lee JH, Kim JY, Noh S, Lee H, Lee SY, Mun JY, Park H, Chung WS (2021). Astrocytes phagocytose adult hippocampal synapses for circuit homeostasis. Nature.

[R96] Lehrman EK, Wilton DK, Litvina EY, Welsh CA, Chang ST, Frouin A, Walker AJ, Heller MD, Umemori H, Chen C, Stevens B (2018). CD47 protects synapses from excess microglia-mediated pruning during development. Neuron.

[R97] Lemke G (2019). How macrophages deal with death. Nat Rev Immunol.

[R98] Li J, Zhao Y, Wang N (2023). Physiological and pathological functions of TMEM30A: an essential subunit of P4-ATPase phospholipid flippases. J Lipids.

[R99] Li T, Chiou B, Gilman CK, Luo R, Koshi T, Yu D, Oak HC, Giera S, Johnson-Venkatesh E, Muthukumar AK, Stevens B, Umemori H, Piao X (2020). A splicing isoform of GPR56 mediates microglial synaptic refinement via phosphatidylserine binding. EMBO J.

[R100] Li T, Yu D, Oak HC, Zhu B, Wang L, Jiang X, Molday RS, Kriegstein A, Piao X (2021). Phospholipid-flippase chaperone CDC50A is required for synapse maintenance by regulating phosphatidylserine exposure. Embo j.

[R101] Li T, Luo R, Schmidt R, D’Alessandro N, Kishore P, Zhu B, Yu D, Piao X (2023). GPR56 S4 variant is required for microglia-mediated synaptic pruning. Glia.

[R102] Li X, Johann S, Rune GM, Bender RA (2021). Sex-specific regulation of spine density and synaptic proteins by G-protein-coupled estrogen receptor (GPER)1 in developing hippocampus. Neuroscience.

[R103] Liao D, Hessler NA, Malinow R (1995). Activation of postsynaptically silent synapses during pairing-induced LTP in CA1 region of hippocampal slice. Nature.

[R104] Lo LH, Lai KO (2020). Dysregulation of protein synthesis and dendritic spine morphogenesis in ASD: studies in human pluripotent stem cells. Mol Autism.

[R105] Maejima Y, Horita S, Yokota S, Yamachi M, Shimizu M, Ono T, Yu Z, Tomita H, Shimomura K (2022). Surface translocation of Kir2.1 channel induces IL-1β secretion in microglia. Mol Cell Neurosci.

[R106] Manich G, Recasens M, Valente T, Almolda B, González B, Castellano B (2019). Role of the CD200-CD200R axis during homeostasis and neuroinflammation. Neuroscience.

[R107] Michalska JM, Lyudchik J, Velicky P, Štefaničková H, Watson JF, Cenameri A, Sommer C, Amberg N, Venturino A, Roessler K, Czech T, Höftberger R, Siegert S, Novarino G, Jonas P, Danzl JG (2024). Imaging brain tissue architecture across millimeter to nanometer scales. Nat Biotechnol.

[R108] Min X, Wang JY, Zong FJ, Zhao J, Liu N, He KW (2023). miR-34a regulates silent synapse and synaptic plasticity in mature hippocampus. Prog Neurobiol.

[R109] Mou TM, Lane MV, Ireland DDC, Verthelyi D, Tonelli LH, Clark SM (2022). Association of complement component 4 with neuroimmune abnormalities in the subventricular zone in schizophrenia and autism spectrum disorders. Neurobiol Dis.

[R110] Moulin TC, Rayêe D, Schiöth HB (2022). Dendritic spine density changes and homeostatic synaptic scaling: a meta-analysis of animal studies. Neural Regen Res.

[R111] Müller NIC, Sonntag M, Maraslioglu A, Hirtz JJ, Friauf E (2019). Topographic map refinement and synaptic strengthening of a sound localization circuit require spontaneous peripheral activity. J Physiol.

[R112] Nguyen PT, Dorman LC, Pan S, Vainchtein ID, Han RT, Nakao-Inoue H, Taloma SE, Barron JJ, Molofsky AB, Kheirbek MA, Molofsky AV (2020). Microglial remodeling of the extracellular matrix promotes synapse plasticity. Cell.

[R113] Nisar S, Bhat AA, Masoodi T, Hashem S, Akhtar S, Ali TA, Amjad S, Chawla S, Bagga P, Frenneaux MP, Reddy R, Fakhro K, Haris M (2022). Genetics of glutamate and its receptors in autism spectrum disorder. Mol Psychiatry.

[R114] Nishibe M, Toyoda H, Hiraga SI, Yamashita T, Katsuyama Y (2022). Synaptic and genetic bases of impaired motor learning associated with modified experience-dependent cortical plasticity in heterozygous reeler mutants. Cereb Cortex.

[R115] Pagani M, Barsotti N, Bertero A, Trakoshis S, Ulysse L, Locarno A, Miseviciute I, De Felice A, Canella C, Supekar K, Galbusera A, Menon V, Tonini R, Deco G, Lombardo MV, Pasqualetti M, Gozzi A (2021). mTOR-related synaptic pathology causes autism spectrum disorder-associated functional hyperconnectivity. Nat Commun.

[R116] Paolicelli RC, Bolasco G, Pagani F, Maggi L, Scianni M, Panzanelli P, Giustetto M, Ferreira TA, Guiducci E, Dumas L, Ragozzino D, Gross CT (2011). Synaptic pruning by microglia is necessary for normal brain development. Science.

[R117] Park J, Choi Y, Jung E, Lee SH, Sohn JW, Chung WS (2021). Microglial MERTK eliminates phosphatidylserine-displaying inhibitory post-synapses. EMBO J.

[R118] Park KK, Liu K, Hu Y, Smith PD, Wang C, Cai B, Xu B, Connolly L, Kramvis I, Sahin M, He Z (2008). Promoting axon regeneration in the adult CNS by modulation of the PTEN/mTOR pathway. Science.

[R119] Peters A, Reisch C, Langemann D (2018). LTP or LTD? Modeling the influence of stress on synaptic plasticity. eNeuro.

[R120] Piochon C, Kano M, Hansel C (2016). LTD-like molecular pathways in developmental synaptic pruning. Nat Neurosci.

[R121] Popescu AS, Butler CA, Allendorf DH, Piers TM, Mallach A, Roewe J, Reinhardt P, Cinti A, Redaelli L, Boudesco C, Pradier L, Pocock JM, Thornton P, Brown GC (2023). Alzheimer’s disease-associated R47H TREM2 increases, but wild-type TREM2 decreases, microglial phagocytosis of synaptosomes and neuronal loss. Glia.

[R122] Pumo GM, Kitazawa T, Rijli FM (2022). Epigenetic and transcriptional regulation of spontaneous and sensory activity dependent programs during neuronal circuit development. Front Neural Circuits.

[R123] Purves D, Lichtman JW (1980). Elimination of synapses in the developing nervous system. Science.

[R124] Qi Y, Zhou Y, Li J, Zhu F, Guo G, Wang C, Yu M, Wang Y, Ma T, Feng S, Zhou L (2024). 3’-Deoxyadenosin alleviates methamphetamine-induced aberrant synaptic plasticity and seeking behavior by inhibiting the NLRP3 inflammasome. Neural Regen Res.

[R125] Qian H, Gao F, Wu X, Lin D, Huang Y, Chen A, Deng J, Gong C, Chen X, Zheng X (2023). Activation of the CD200/CD200R1 axis attenuates neuroinflammation and improves postoperative cognitive dysfunction via the PI3K/Akt/NF-κB signaling pathway in aged mice. Inflamm Res.

[R126] Reich DS, Lucchinetti CF, Calabresi PA (2018). Multiple sclerosis. N Engl J Med.

[R127] Rizo J (2022). Molecular mechanisms underlying neurotransmitter release. Annu Rev Biophys.

[R128] Rodriguez G, Mesik L, Gao M, Parkins S, Saha R, Lee HK (2019). Disruption of NMDAR function prevents normal experience-dependent homeostatic synaptic plasticity in mouse primary visual cortex. J Neurosci.

[R129] Ronnevi LO, Conradi S (1974). Ultrastructural evidence for spontaneous elimination of synaptic terminals on spinal motoneurons in the kitten. Brain Res.

[R130] Rotterman TM, Akhter ET, Lane AR, MacPherson KP, García VV, Tansey MG, Alvarez FJ (2019). Spinal motor circuit synaptic plasticity after peripheral nerve injury depends on microglia activation and a CCR2 mechanism. J Neurosci.

[R131] Sahasrabuddhe V, Ghosh HS (2022). Cx3Cr1-Cre induction leads to microglial activation and IFN-1 signaling caused by DNA damage in early postnatal brain. Cell Rep.

[R132] Sarn N, Jaini R, Thacker S, Lee H, Dutta R, Eng C (2021). Cytoplasmic-predominant Pten increases microglial activation and synaptic pruning in a murine model with autism-like phenotype. Mol Psychiatry.

[R133] Sawchuk SD, Reid HMO, Neale KJ, Shin J, Christie BR (2020). Effects of ethanol on synaptic plasticity and NMDA currents in the juvenile rat dentate gyrus. Brain Plast.

[R134] Schafer DP, Lehrman EK, Kautzman AG, Koyama R, Mardinly AR, Yamasaki R, Ransohoff RM, Greenberg ME, Barres BA, Stevens B (2012). Microglia sculpt postnatal neural circuits in an activity and complement-dependent manner. Neuron.

[R135] Schartz ND, Tenner AJ (2020). The good, the bad, and the opportunities of the complement system in neurodegenerative disease. J Neuroinflammation.

[R136] Schoonover KE, Miller NE, Fish KN, Lewis DA (2024). Scaling of smaller pyramidal neuron size and lower energy production in schizophrenia. Neurobiol Dis.

[R137] Scott-Hewitt N, Perrucci F, Morini R, Erreni M, Mahoney M, Witkowska A, Carey A, Faggiani E, Schuetz LT, Mason S, Tamborini M, Bizzotto M, Passoni L, Filipello F, Jahn R, Stevens B, Matteoli M (2020). Local externalization of phosphatidylserine mediates developmental synaptic pruning by microglia. EMBO J.

[R138] Sekar A, Bialas AR, de Rivera H, Davis A, Hammond TR, Kamitaki N, Tooley K, Presumey J, Baum M, Van Doren V, Genovese G, Rose SA, Handsaker RE, Daly MJ, Carroll MC, Stevens B, McCarroll SA (2016). Schizophrenia risk from complex variation of complement component 4. Nature.

[R139] Sell GL, Barrow SL, McAllister AK (2024). Glutamate signaling and neuroligin/neurexin adhesion play opposing roles that are mediated by major histocompatibility complex I molecules in cortical synapse formation. J Neurosci.

[R140] Shacham-Silverberg V, Sar Shalom H, Goldner R, Golan-Vaishenker Y, Gurwicz N, Gokhman I, Yaron A (2018). Phosphatidylserine is a marker for axonal debris engulfment but its exposure can be decoupled from degeneration. Cell Death Dis.

[R141] Shen K, Reichelt M, Kyauk RV, Ngu H, Shen YA, Foreman O, Modrusan Z, Friedman BA, Sheng M, Yuen TJ (2021). Multiple sclerosis risk gene Mertk is required for microglial activation and subsequent remyelination. Cell Rep.

[R142] Shi X, Luo L, Wang J, Shen H, Li Y, Mamtilahun M, Liu C, Shi R, Lee JH, Tian H, Zhang Z, Wang Y, Chung WS, Tang Y, Yang GY (2021). Stroke subtype-dependent synapse elimination by reactive gliosis in mice. Nat Commun.

[R143] Shin W, Kim K, Serraz B, Cho YS, Kim D, Kang M, Lee EJ, Lee H, Bae YC, Paoletti P, Kim E (2020). Early correction of synaptic long-term depression improves abnormal anxiety-like behavior in adult GluN2B-C456Y-mutant mice. PLoS Biol.

[R144] Shui M, Sun Y, Lin D, Xue Z, Liu J, Wu A, Wei C (2022). Anomalous levels of CD47/signal regulatory protein alpha in the hippocampus lead to excess microglial engulfment in mouse model of perioperative neurocognitive disorders. Front Neurosci.

[R145] Sidorov MS, Kaplan ES, Osterweil EK, Lindemann L, Bear MF (2015). Metabotropic glutamate receptor signaling is required for NMDA receptor-dependent ocular dominance plasticity and LTD in visual cortex. Proc Natl Acad Sci U S A.

[R146] Singh P, Donlea JM (2020). Bidirectional regulation of sleep and synapse pruning after neural injury. Curr Biol.

[R147] Skoug C, Martinsson I, Gouras GK, Meissner A, Duarte JMN (2022). Sphingosine 1-phoshpate receptors are located in synapses and control spontaneous activity of mouse neurons in culture. Neurochem Res.

[R148] Smucny J, Dienel SJ, Lewis DA, Carter CS (2022). Mechanisms underlying dorsolateral prefrontal cortex contributions to cognitive dysfunction in schizophrenia. Neuropsychopharmacology.

[R149] Somaiya RD, Feller MB (2024). Visualizing synaptic pruning in the mammalian brain. Science.

[R150] Son S, Nagahama K, Lee J, Jung K, Kwak C, Kim J, Noh YW, Kim E, Lee S, Kwon HB, Heo WD (2024). Real-time visualization of structural dynamics of synapses in live cells in vivo. Nat Methods.

[R151] Song X, Li Y, Guo R, Yu Q, Liu S, Teng Q, Chen ZR, Xie J, Gong S, Liu K (2022). Cochlear resident macrophage mediates development of ribbon synapses via CX3CR1/CX3CL1 axis. Front Mol Neurosci.

[R152] Stein IS, Zito K (2019). Dendritic spine elimination: molecular mechanisms and implications. Neuroscientist.

[R153] Stevens B, Allen NJ, Vazquez LE, Howell GR, Christopherson KS, Nouri N, Micheva KD, Mehalow AK, Huberman AD, Stafford B, Sher A, Litke AM, Lambris JD, Smith SJ, John SW, Barres BA (2007). The classical complement cascade mediates CNS synapse elimination. Cell.

[R154] Sun H, He X, Tao X, Hou T, Chen M, He M, Liao H (2020). The CD200/CD200R signaling pathway contributes to spontaneous functional recovery by enhancing synaptic plasticity after stroke. J Neuroinflammation.

[R155] Suzuki J, Imanishi E, Nagata S (2016). Xkr8 phospholipid scrambling complex in apoptotic phosphatidylserine exposure. Proc Natl Acad Sci U S A.

[R156] Tanaka S, Miyashita M, Wakabayashi N, O’Hashi K, Tani T, Ribot J (2020). Development and reorganization of orientation representation in the cat visual cortex: experience-dependent synaptic rewiring in early life. Front Neuroinform.

[R157] Tang G, Gudsnuk K, Kuo SH, Cotrina ML, Rosoklija G, Sosunov A, Sonders MS, Kanter E, Castagna C, Yamamoto A, Yue Z, Arancio O, Peterson BS, Champagne F, Dwork AJ, Goldman J, Sulzer D (2014). Loss of mTOR-dependent macroautophagy causes autistic-like synaptic pruning deficits. Neuron.

[R158] Teichert M, Liebmann L, Hübner CA, Bolz J (2017). Homeostatic plasticity and synaptic scaling in the adult mouse auditory cortex. Sci Rep.

[R159] Thomazeau A, Bosch M, Essayan-Perez S, Barnes SA, De Jesus-Cortes H, Bear MF (2021). Dissociation of functional and structural plasticity of dendritic spines during NMDAR and mGluR-dependent long-term synaptic depression in wild-type and fragile X model mice. Mol Psychiatry.

[R160] Tononi G, Cirelli C (2014). Sleep and the price of plasticity: from synaptic and cellular homeostasis to memory consolidation and integration. Neuron.

[R161] Tuan LH, Lee LJ (2019). Microglia-mediated synaptic pruning is impaired in sleep-deprived adolescent mice. Neurobiol Dis.

[R162] Tuan LH, Tsao CY, Lee LJ, Lee LJ (2021). Voluntary exercise ameliorates synaptic pruning deficits in sleep-deprived adolescent mice. Brain Behav Immun.

[R163] Urrutia-Ruiz C, Rombach D, Cursano S, Gerlach-Arbeiter S, Schoen M, Bockmann J, Demestre M, Boeckers TM (2022). Deletion of the autism-associated protein SHANK3 abolishes structural synaptic plasticity after brain trauma. Int J Mol Sci.

[R164] Vainchtein ID, Chin G, Cho FS, Kelley KW, Miller JG, Chien EC, Liddelow SA, Nguyen PT, Nakao-Inoue H, Dorman LC, Akil O, Joshita S, Barres BA, Paz JT, Molofsky AB, Molofsky AV (2018). Astrocyte-derived interleukin-33 promotes microglial synapse engulfment and neural circuit development. Science.

[R165] Wang C, Yue H, Hu Z, Shen Y, Ma J, Li J, Wang XD, Wang L, Sun B, Shi P, Wang L, Gu Y (2020). Microglia mediate forgetting via complement-dependent synaptic elimination. Science.

[R166] Wang CS, Kavalali ET, Monteggia LM (2022). BDNF signaling in context: From synaptic regulation to psychiatric disorders. Cell.

[R167] Wang G, An T, Lei C, Zhu X, Yang L, Zhang L, Zhang R (2022). Antidepressant-like effect of ginsenoside Rb1 on potentiating synaptic plasticity via the miR-134-mediated BDNF signaling pathway in a mouse model of chronic stress-induced depression. J Ginseng Res.

[R168] Wang H, Liu H, Zhang ZW (2011). Elimination of redundant synaptic inputs in the absence of synaptic strengthening. J Neurosci.

[R169] Wang L, Ling H, He H, Hu N, Xiao L, Zhang Y, Xie L, You Z (2023). Dysfunctional synaptic pruning by microglia correlates with cognitive impairment in sleep-deprived mice: Involvement of CX3CR1 signaling. Neurobiol Stress.

[R170] Wang Y, Fu WY, Cheung K, Hung KW, Chen C, Geng H, Yung WH, Qu JY, Fu AKY, Ip NY (2021). Astrocyte-secreted IL-33 mediates homeostatic synaptic plasticity in the adult hippocampus. Proc Natl Acad Sci U S A.

[R171] Wang Z, Fan J, Wang J, Li Y, Duan D, Du G, Wang Q (2016). Chronic cerebral hypoperfusion induces long-lasting cognitive deficits accompanied by long-term hippocampal silent synapses increase in rats. Behav Brain Res.

[R172] Werneburg S, Jung J, Kunjamma RB, Ha SK, Luciano NJ, Willis CM, Gao G, Biscola NP, Havton LA, Crocker SJ, Popko B, Reich DS, Schafer DP (2020). Targeted complement inhibition at synapses prevents microglial synaptic engulfment and synapse loss in demyelinating disease. Immunity.

[R173] Whitt JL, Petrus E, Lee HK (2014). Experience-dependent homeostatic synaptic plasticity in neocortex. Neuropharmacology.

[R174] Wiegert JS, Oertner TG (2013). Long-term depression triggers the selective elimination of weakly integrated synapses. Proc Natl Acad Sci U S A.

[R175] Wiesel TN, Hubel DH (1963). Single-cell responses in striate cortex of kittens deprived of vision in one eye. J Neurophysiol.

[R176] Wilkerson JR, Tsai NP, Maksimova MA, Wu H, Cabalo NP, Loerwald KW, Dictenberg JB, Gibson JR, Huber KM (2014). A role for dendritic mGluR5-mediated local translation of Arc/Arg3.1 in MEF2-dependent synapse elimination. Cell Rep.

[R177] Wilton DK, Dissing-Olesen L, Stevens B (2019). Neuron-glia signaling in synapse elimination. Annu Rev Neurosci.

[R178] Woo NH, Teng HK, Siao CJ, Chiaruttini C, Pang PT, Milner TA, Hempstead BL, Lu B (2005). Activation of p75NTR by proBDNF facilitates hippocampal long-term depression. Nat Neurosci.

[R179] Wu Q, Wang H, Liu X, Zhao Y, Su P (2023). Microglial activation and over pruning involved in developmental epilepsy. J Neuropathol Exp Neurol.

[R180] Wu T (2019). Complement C3 is activated in human AD brain and is required for neurodegeneration in mouse models of amyloidosis and tauopathy. Cell Rep.

[R181] Xin D, Li T, Chu X, Ke H, Liu D, Wang Z (2021). MSCs-extracellular vesicles attenuated neuroinflammation, synapse damage and microglial phagocytosis after hypoxia-ischemia injury by preventing osteopontin expression. Pharmacol Res.

[R182] Xu F, Han L, Wang Y, Deng D, Ding Y, Zhao S, Zhang Q, Ma L, Chen X (2023). Prolonged anesthesia induces neuroinflammation and complement-mediated microglial synaptic elimination involved in neurocognitive dysfunction and anxiety-like behaviors. BMC Med.

[R183] Xu X, Zhang H, Li J, Chen Y, Zhong W, Chen Y, Ma X (2023). Combination of EPC-EXs and NPC-EXs with miR-126 and miR-210 overexpression produces better therapeutic effects on ischemic stroke by protecting neurons through the Nox2/ROS and BDNF/TrkB pathways. Exp Neurol.

[R184] Yao M, Meng M, Yang X, Wang S, Zhang H, Zhang F, Shi L, Zhang Y, Zhang X, Xu Z (2022). POSH regulates assembly of the NMDAR/PSD-95/Shank complex and synaptic function. Cell Rep.

[R185] Yilmaz M, Yalcin E, Presumey J, Aw E, Ma M, Whelan CW, Stevens B, McCarroll SA, Carroll MC (2021). Overexpression of schizophrenia susceptibility factor human complement C4A promotes excessive synaptic loss and behavioral changes in mice. Nat Neurosci.

[R186] Yin XL, Jie HQ, Liang M, Gong LN, Liu HW, Pan HL, Xing YZ, Shi HB, Li CY, Wang LY, Yin SK (2018). Accelerated development of the first-order central auditory neurons with spontaneous activity. Front Mol Neurosci.

[R187] Yuan T, Orock A, Greenwood-VanMeerveld B (2022). An enriched environment reduces chronic stress-induced visceral pain through modulating microglial activity in the central nucleus of the amygdala. Am J Physiol Gastrointest Liver Physiol.

[R188] Zhang C, Yadav S, Speer CM (2023). The synaptic basis of activity-dependent eye-specific competition. Cell Rep.

[R189] Zhang C, Qi H, Jia D, Zhao J, Xu C, Liu J, Cui Y, Zhang J, Wang M, Chen M, Tang B (2024). Cognitive impairment in Alzheimer’s disease FAD(4T) mouse model: Synaptic loss facilitated by activated microglia via C1qA. Life Sci.

[R190] Zhang H, Jiang X, Ma L, Wei W, Li Z, Chang S, Wen J, Sun J, Li H (2022). Role of Aβ in Alzheimer’s-related synaptic dysfunction. Front Cell Dev Biol.

[R191] Zhang J, Wang J, Xu X, You Z, Huang Q, Huang Y, Guo Q, Guan Y, Zhao J, Liu J, Xu W, Deng Y, Xie F, Li B (2023). In vivo synaptic density loss correlates with impaired functional and related structural connectivity in Alzheimer’s disease. J Cereb Blood Flow Metab.

[R192] Zhang KX, Zhao JJ, Chai W, Chen JY (2021). Synaptic remodeling in mouse motor cortex after spinal cord injury. Neural Regen Res.

[R193] Zhang YQ, Lin WP, Huang LP, Zhao B, Zhang CC, Yin DM (2021). Dopamine D2 receptor regulates cortical synaptic pruning in rodents. Nat Commun.

[R194] Zhang Z, Mao Y, Huang S, Xu R, Huang Y, Li S, Sun Y, Gu X, Ma Z (2024). Microglia promote inhibitory synapse phagocytosis in the spinal cord dorsal horn and modulate pain-like behaviors in a murine cancer-induced bone pain model. Anesth Analg.

[R195] Zhao Y, Zhang J, Cheng X, Huang W, Shen S, Wu S, Huang Y, Nie G, Wang H, Qiu W (2023). Targeting L-selectin lymphocytes to deliver immunosuppressive drug in lymph nodes for durable multiple sclerosis treatment. Adv Sci (Weinh).

[R196] Zheng L, Wang Y, Shao B, Zhou H, Li X, Zhang C, Sun N, Shi J (2022). Multiple mild stimulations reduce membrane distribution of CX3CR1 promoted by Annexin a1 in microglia to attenuate excessive dendritic spine pruning and cognitive deficits caused by a transient ischemic attack in mice. Neurosci Bull.

[R197] Zhong L, Sheng X, Wang W, Li Y, Zhuo R, Wang K, Zhang L, Hu DD, Hong Y, Chen L, Rao H, Li T, Chen M, Lin Z, Zhang YW, Wang X, Yan XX, Chen X, Bu G, Chen XF (2023). TREM2 receptor protects against complement-mediated synaptic loss by binding to complement C1q during neurodegeneration. Immunity.

